# Revision of Gymnomitriaceae (Marchantiophyta) in the Korean Peninsula

**DOI:** 10.3897/phytokeys.176.62552

**Published:** 2021-04-16

**Authors:** Vadim Bakalin, Seung Se Choi, Seung Jin Park

**Affiliations:** 1 Botanical Garden-Institute, Vladivostok, 690024, Russia Botanical Garden-Institute Vladivostok Russia; 2 Department of Natural Environment Research, National Institute of Ecology, Seocheon, Chungcheongnam-do, 33657, South Korea National Institute of Ecology Seocheon Republic of Korea; 3 Department of Biological Sciences, Jeonbuk National University, Jeonju, Jeollabuk-do, 54896, South Korea Jeonbuk National University Jeonju Republic of Korea

**Keywords:** Gymnomitriaceae, *
Gymnomitrion
*, Hepaticae, Korean Peninsula, *
Marsupella
*, taxonomy

## Abstract

This paper provides a revision of *Gymnomitrion* and *Marsupella* in the Korean Peninsula based on a study of the collections housed in the herbaria of Jeonbuk National University (JNU) and the Botanical Garden-Institute in Vladivostok (VBGI). In total, 12 species were recorded (six in *Gymnomitrion* and seven in *Marsupella*), including four taxa whose identity was not confirmed with the available materials and suspected to be recorded wrongly. Each confirmed species is annotated by morphological descriptions based on available Korean material, data on ecology, distribution, specimens examined as well as illustrations.

## Introduction

Gymnomitriaceae in the recent World Liverwort Checklist (Söderström et al. 2016) include *Nardia*, which is clearly distinct from other genera in the family due to the presence of underleaves. In fact, as demonstrated by Shaw et al. (2015), who were the first to transfer *Nardia* to Gymnomitriaceae, *Nardia* formed a basal clade to all other genera traditionally included in the family (*Gymnomitrion*, *Marsupella*, *Prasanthus*, *Poeltia*). It is therefore questionable whether *Nardia* should be treated as a member of Gymnomitriaceae or as a representative of its own family which has not yet been described. Following the second possibility (although refraining from any taxonomical formalities), we treat Gymnomitriaceae in the Korean Peninsula as housing only *Gymnomitrion* and *Marsupella*, putting aside *Nardia*. This point of view corroborates with that accepted in the worldwide compendium of Gymnomitriaceae by Váňa et al. (2010). Gymnomitriaceae house 85 taxa that occur in all continents. This family is most common and diverse in hemiarctic and alpine areas of Holarctic, with other noticeable diversity centers in the Sino-Himalayas and East Asia (Váňa et al. 2010). The recent advances in the *Marsupella* taxonomy in East Asia revealed that some additional taxa need to be recognized, including one taxon described from the Korean Peninsula that at this stage of knowledge is endemic to the Korean liverwort flora (Bakalin et al. 2019). Temperate East Asia (including Northeast China, Japan, Korean Peninsula and South Kurils) is characterized by the occurrence of a number of remarkable *Marsupella* species with ‘scapanioid’ appearance due to strong distichous leaf arrangement and conduplicate leaves with commonly unequal leaf lobes and distinct keel. These ‘scapanioid’ *Marsupella* are absent outside East Asia. Three species of this group occur in the Korean Peninsula.

The main goal of the present paper was to revise Gymnomitriaceae for the Korean Peninsula as it was never substantially revised. Moreover, despite the peculiarity of Gymnomitriaceae flora in East Asia, the family was never revised in eastern China, adjacent to the Korean Peninsula, while the revision for Japan (Kitagawa 1963) is somewhat outdated. Recent advances in knowledge about Gymnomitriaceae have been summarized in the Liverwort and Hornwort Flora of Korea (Choi et al. 2017). However, the long history of the preparation of this flora has resulted in the fact that it is already out of date, despite being recently published. The flora was based on the data available until the spring of 2015 and does not include novelties published in 2016 and later. Due to these circumstances, it does not include the recently described *Marsupella koreana* Bakalin et Fedosov, which is mistakenly named as *M. pseudofunckii* S.Hatt. in the flora. It also does not include a record of *M. vermiformis* (R.M.Schust.) Bakalin et Fedosov identified by molecular methods (Bakalin et al. 2019). *Gymnomitrion faurianum* (Steph.) Horik. was completely confused with *G. concinnatum* (Lightf.) Corda, while *G. parvitextum* (Steph.) Mamontov, Konstant. et Potemkin was misidentified as *G. commutatum* (Limpr.) Schiffn. It is worth mentioning that *G. commutatum* was nevertheless found in South Korea in Jeju-do, from where it was first reported by Mamontov et al. (2018), whereas other reports of *G. commutatum* occurring in the literature are based entirely on *G. parvitextum*. Therefore, about half of the information provided by Choi et al. (2017) is incorrect. Moreover, the flora provides the only identification keys (unreliable due to the aforementioned omissions) and morphological descriptions but does not include illustrations and taxonomical comments describing differentiation features. These facts inspired us to compile a new version of the Gymnomitriaceae revision.

## Methods

Owing to the data at hand, it is known that Gymnomitriaceae s. str. is represented in the Korean Peninsula by only two genera: *Marsupella* and *Gymnomitrion*. Therefore, regarding the species concept in particular cases, we were guided by recent works in this field, including Bakalin (2016), Bakalin et al. (2019), and Mamontov et al. (2018, 2019). The foundation of the work was formed on the basis of a study of over 500 specimens. This exhausts approximately 90% of all existing specimens of Gymnomitriaceae collected in the Korean peninsula (the numerical data on the northern part of the peninsula are unavailable and not included in this account). The main collections of Korean liverworts are now in JNU and VBGI, although some historical collections are available from HIRO, NICH, and G. Types were studied for several taxa indicated in the text.

**Figure 1.**
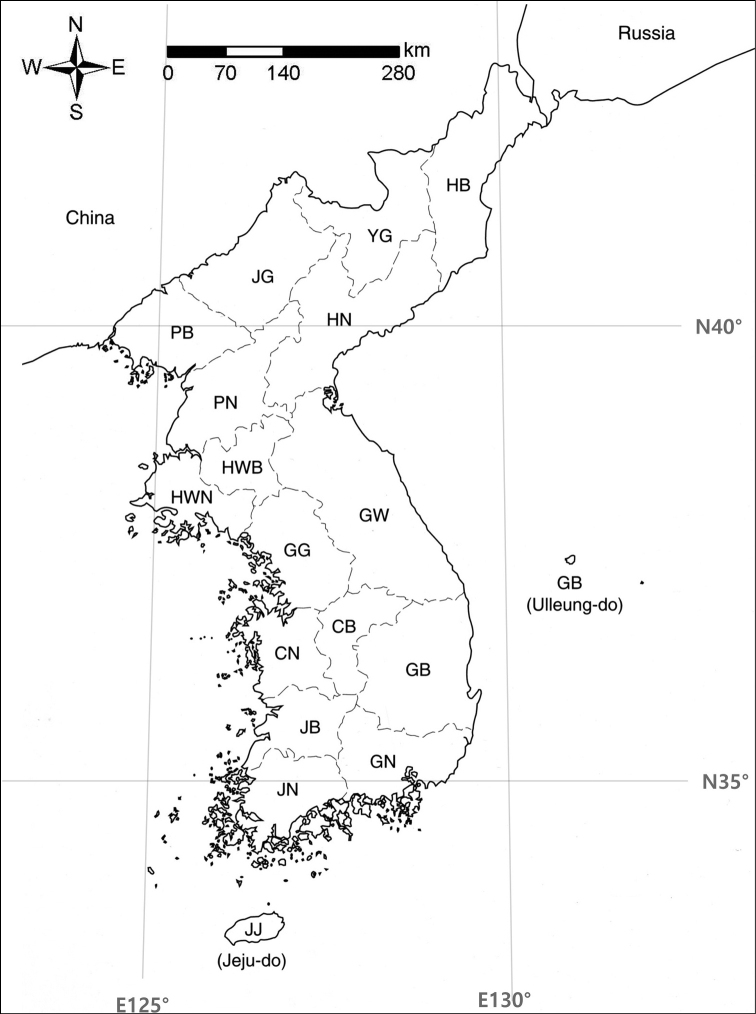
The regionalization (provinces) of the Korean Peninsula accepted in the present paper. Abbreviations: **CB** Chungcheongbuk-do **CN** Chungcheongnam-do **GB** Gyeongsangbuk-do **GN** Gyeongsangnam-do **GG** Gyeonggi-do **GW** Gangwon-do **HB** Hamgyeongbuk-do **HN** Hamgyeongnam-do **HWN** Hwanghaenam-do **HWB** Hwanghaebuk-do **JB** Jeollabuk-do **JG** Jagang-do **JN** Jeollanam-do **JJ** Jeju-do **PB** Pyeonganbuk-do **PN** Pyeongannam-do **YG** Yanggang-do.

This paper provides the descriptions compiled based on the study of specimens collected in Korea and to a lesser extent, the types. The morphological descriptions are supplemented by figures, list of specimens examined, discussion on ecology, distribution, and, in some cases, the taxonomy and morphology. The distribution within the Korean Peninsula, described using official regionalization, extended throughout the Korean Peninsula despite covering different countries (Fig. 1). This regionalization was accepted by both North Korean bryologists (Kim and Hwang 1991) and South Korean and Japanese hepaticologists (Yamada and Choe 1997) and is the most appropriate for our tasks. The data on the species distribution in adjacent areas are mostly from Bakalin (2010: the Russian Far East), Yamada and Iwatsuki (2006: Japan), Piippo (1990: China), the recent updates were added from the literature cited in each case separately.

The morphological descriptions for family and genera use features known in the taxa recorded in the Korean Peninsula. The taxa reported in the literature, but not revealed in the present revision (and suspected to be erroneously reported) are keyed out, in square brackets and are not supplied with descriptions; rather they are briefly discussed in the section ‘Excluded taxa’. After the accepted name of the species, only a few common synonyms are provided, with an emphasis on names previously applied to plants from the Korean Peninsula.

## Taxonomic treatment

### Gymnomitriaceae

Taxon classificationPlantaeJungermannialesGymnomitriaceae

H. Klinggr., Höh. Crypt. Preuss.: 16, 1858.

0F500F47-3FD0-56F0-8957-8ED9B4DD67E8

#### Description.

Plants rigid to soft, variously colored. Rhizoids sparse to dense, mostly colorless, rarely purple. Stem with differentiation into scleroderm, hyaloderm and inner tissue or without differentiation (then mostly pachydermous in structure). Leaves shallowly emarginate to bilobed (not more than 2/5 of leaf length) into equal to unequal lobes. Underleaves absent (present in *Nardia* not treated here). Androecia intercalary, stalk biseriate. Perianth well developed to reduced or totally absent. Perigynium well developed to absent (and then perianth wanting or strongly reduced). Elaters with 2–4 spirals.

#### Comment.

The above description is applicable to the Gymnomitriaceae taxa occurring in the Korean Peninsula.

### Key to genera recorded in Korea

**Table:** 

1	Plants with well-developed perigynium and reduced, but always distinct perianth, loosely leaved, leaves mostly (excluding androecious branches) not imbricate, if imbricate (*M. vermiformis*), then leaves narrower than the stem; leaf margin plane or revolute, if revolute the cell size in the midleaf wider 10 μm wide and never with large subquadrate trigones roughly equal in size to the cell lumen, giving the cell network a chequered appearance	*** Marsupella ***
–	Plants with strongly reduced to absent perigynium and virtually absent perianth, leaves wider than stem, with margin plane or revolute, commonly imbricate, if not (*G. parvitextum*) then leaf margin is narrowly revolute (at least evident in the leaf base), cells in the midleaf less 10 μm wide, with large subquadrate trigones roughly equal in size to the cell lumen, giving the cell network a chequered appearance	*** Gymnomitrion ***

### Marsupella

Taxon classificationPlantaeJungermannialesGymnomitriaceae

Dumort., Commentat. Bot. (Dumortier): 114, 1822.

B9DBF1A9-3238-5605-B9DE-23DA6E209D18

#### Description.

Plants forming loose patches, commonly ascending or rarely creeping in habitats with strong insolation and incrusted by soil particles, deeply (deep green, brown-green, brown purple, blackish brown) to pale (yellowish, brownish, greenish and their combinations) colored, merely rigid, varying in size from 0.5 to 2.0 mm wide and 5.0–50.0 mm long. Rhizoids sporadic to solitary, although invariably present in ventral geotropic leafless stolons, colorless to grayish and soft-textured, or rarely and solitary purple and rigid. Stem with common ventral branching and rare lateral branches, with characteristic geotropic stolons present in the majority of taxa; in cross section mostly differentiated into three strata: hyaloderm, scleroderm and inner tissue. Leaves transversely or nearly so inserted, obliquely to erect spreading from sheathing or not sheathing base, concave to canaliculate and strongly conduplicate, divided by evident but not deep sinus into two equal to strongly unequal lobes with rounded to acute apices. Underleaves absent. Cells in the leaf in the most taxa pachydermous with large convex trigones, unequally thickened along margin and having smooth cuticle; oil-bodies few in number, (1–)2–3(–4) per cell, finely granulate to papillose or almost smooth, rarely with central eye. Dioicous. Androecia intercalary, spicate, antheridium stalk biseriate (rarely uniseriate near the base). Perianth short, but always developed, onion-shaped or conical, wider than long, mostly hidden, but rarely emergent from bracts; perigynium well developed, commonly 2–3 times longer than the perianth, with (1–)2 pairs of bracts. Elaters 2–4-spiral, spores brownish, papillose.

#### Comment.

*Marsupella* is easily recognized, even in the field, due to characteristic rigid texture, transversely inserted and sheathing stem leaves, absence of underleaves, and hidden perianth. Under the microscope, additional features such as pachydermous leaf cells, defined scleroderm in stem cross section, few oil bodies, and high perigynium are helpful to refer specimens to this genus.

This paper accepts recent emendations for the circumscription of *Marsupella*, the most valuable being the removal of ‘perianth-less’ taxa (e.g., ‘*M. commutata*’) to *Gymnomitrion*. Five species were confirmed for this genus in Korean flora and one (*M. sphacelata*) regarded as likely reported erroneously.

In the specimens examined section, we cite only one specimen per locality (or 2 to 3, if they were collected at different elevations) with the intention of economizing space. Only specimens from the Korean Peninsula are cited.

### Key to *Marsupella* species recorded in Korea

**Table:** 

1	Plants with strongly distichously arranged and keeled to narrowly canaliculate leaves, lobe apices acute to obtuse	**2**
–	Plants not strongly distichous, concave to canaliculate (never conduplicate), lobe apices acute to rounded	**4**
2	Plants noticeable dilated to perianth, leaves conduplicate, lobes unequal, with margin plane, leaf keel distinct	***M. pseudofunckii***
–	Plants slightly or not dilated to the perianth, conduplicate to canaliculate, lobes unequal to subequal, with margin plane to recurved, leaf keel distinct or not	**3**
3	Leaf lobes subequal, leaf margin commonly undulate and never narrowly recurved, leaf lobes commonly turned to dorsal side, midleaf cell with thick walls and relatively small and concave trigones	***M. yakushimensis***
–	Leaf lobes unequal, leaf margin recurved, never undulate, leaf lobes never turned dorsally, midleaf cells with thin to slightly thickened walls and moderate to large, convex trigones	***M. koreana***
4	Plants vermiform, with leaves narrower than the stem (except very apex) and very tightly appressed to the stem	***M. vermiformis***
–	Plants with leaves spreading, distinctly wider than the stem	**5**
5	Leaves lax, sometimes shallowly undulate at margin, divided by sinus descending at most for 2/5 of the leaf length, plants with green to brownish coloration (never red), hyaloderm in stem cross section with cells twice as large as inner cells, oil bodies not biconcentric [not confirmed for Korea]	[***M. sphacelata***]
–	Leaves merely rigid, with recurved margins (sometimes obscurely so or only near the leaf base), distinctly concave, never undulate at margins, divided by sinus descending at most for 1/5(-1/4) of the leaf length, plants sometimes red, hyaloderm cells in the stem cross section less 1.5 times as inner cells, oil bodies biconcentric or not	**6**
6	Oil bodies without central eye or with very small eye, plants’ color varying in exposed places to brown and rusty, but never red and purple	***M. apertifolia***
–	Oil bodies always or at least in 50% of oil bodies with central eye, plants commonly purple in exposed places	***M. tubulosa***

### Marsupella apertifolia

Taxon classificationPlantaeJungermannialesGymnomitriaceae

Steph., Bull. Herb. Boiss. ser. 2, 1: 162 (Spec. Hep. 2: 23), 1901

2BD05DF8-300B-5EFC-9BCC-41AF4E7B362E

Figure 2

Marsupella emarginata subsp. tubulosa var. apertifolia (Steph.) N.Kitag., J. Hattori Bot. Lab. 26: 89, 1963.
Marsupella tubulosa var. apertifolia (Steph.) S.Hatt., Bull. Tokyo Sci. Mus. 11: 78, 1944.

#### Type.

Japan, Miyokosan, U. Faurie 75 (***lectotype*** (designated here): G [009469!]).

#### Description.

Plants in rather loose patches, rigid to more soft, erect or nearly so, mostly deep green to brownish green in color, but with many other variants intergrading to yellowish-brownish, yellowish, and pale greenish (later only in shaded wet places) or to deep brown and rusty pigmentation in insolated moist habitats; mostly 1.2–1.6 mm wide and 7.0–15.0 mm long, with small forms starting from 0.75–1.0 mm wide and robust varying to 1.5–2.2 mm wide. Branching lateral (rare) or ventral as subfloral innovations (more common), stem transversely elliptic in the cross section, 200.0–300.0 μm high (extreme variants not included) and 250.0–350.0 μm wide; outer cells (hyaloderm) nearly thin, with small trigones, 15.0–25.0 μm along margin, scleroderm in 3–4 layers, with cells slightly smaller, very thick-walled, with lumen just 8.0–11.0 μm, inner cells (10.0–)12.0–20.0 μm, thin- to slightly thickened, with moderate in size, triangle to concave trigones. Rhizoids sparse to virtually absent, mostly colorless to brownish, in unclear obliquely to erect spreading fascicles, rarely (and very few in number) separated and deep purple. Leaves mostly contiguous and loosely enclosed one to another, to subimbricate or nearly distant in lax modifications, concave to canaliculate-concave, transversely inserted, evidently sheathing the stem in the base and obliquely to erect spreading above, transversely, subtransversely or (more rarely) obliquely oriented, with margin commonly narrowly recurved, at least in lower half of the leaf; transversely elliptic to orbicular and widely ovate in shape, mostly 500.0–750.0 μm long and 550.0–1050.0 μm wide, reaching in lax forms 1500.0–2250.0 × 1550.0–2750.0 μm, divided by sinus descending to 1/7–1/5(1/4) of leaf length into two nearly equal to subequal lobes; sinus varying from narrowly to widely **γ**-shaped; lobes rounded to (rarely) obtuse in apex. Cells in the midleaf subisodiametric to shortly oblong, (12.0–)20.0–25.0 × (12.0–)13.0–25.0 μm, thin-walled to slightly thickened, trigones mostly large, rarer moderate in size, convex to bulging, cuticle smooth; cells along margin in upper part of leaf 5.0–8.0(–10.0) μm, mostly unequally thickened due to trigones confluence, trigones large, convex to concave, cuticle smooth; cells in the lobe middle similar to that in the midleaf or slightly smaller, 12.0–20.0 × 11.0–15.0 μm, thin-walled, trigones large, convex to bulging, cuticle smooth. Dioicous. Androecia intercalary, spicate, with 3–4 pairs of bracts, (1–)2–3-androus, antheridium body obovate, 130.0–150.0 μm wide, stalk biseriate, 100–150 μm long; bracts strongly inflate in lower half and obliquely to erect (especially lobes) spreading above, trapezoidal-subtransversely elliptic. Perianth hidden within bracts or very shortly exerted, conical to onion-shaped, 400.0–750.0 × 750.0 μm; perigynium 750.0–1000.0 μm long, with two pairs of bracts; bracts sheathing perigynium in lower part and obliquely spreading above (lobes of lower pair commonly deflexed).

**Figure 2.**
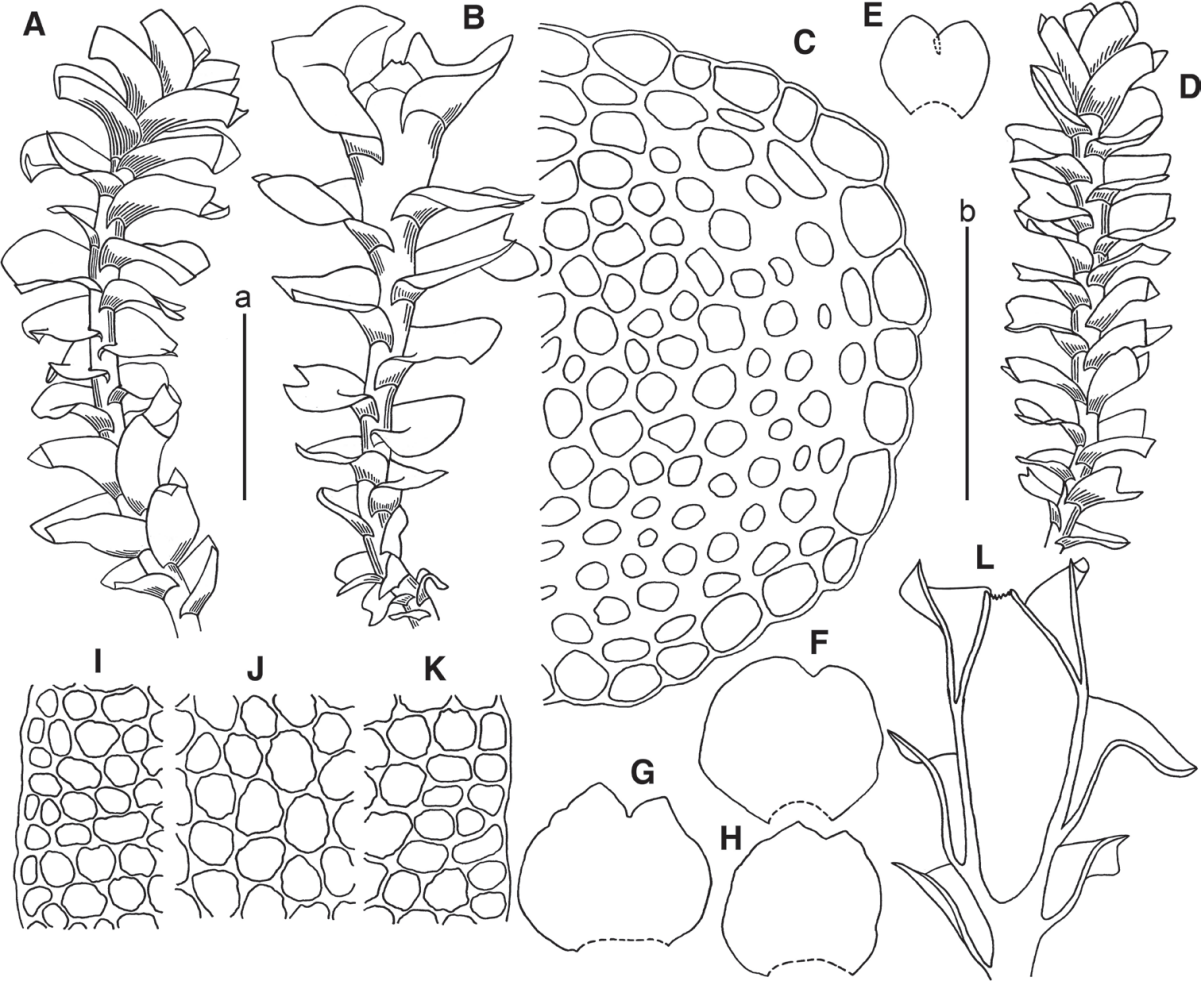
*Marsupella apertifolia* Steph. **A** male plant **B** female plants **C** stem cross section (fragment) **D** plant habit **E–H** leaves **I, K** cells along leaf margin **J** midleaf cells **L** gynoecium longitudinal section. Scale bars: a 1 mm (**A, B, D–H, L**); b 100 µm (**C, I–K**). All from *Choi 7383* (JNU). Drawing by S.J. Park.

#### Ecology.

Acidophilic hygro- to hydrophyte, occupying various habitats, from very wet (and even submerged) shaded cliffs near running water to moist mineral substrata in full sun. In moist and sunny habitats, robust phases are formed (then commonly acquiring deep rusty-brown pigmentation), where it is associated with *Anastrophyllum assimile*, *Trilophozia quinquedentata*, and *Diplophyllum taxifolium*. As an extreme variant, the species may be intermixed with *Gymnomitrion faurianum*. In wet and shady habitats, its common association is Cephalozia otaruensis^*^.

#### Distribution.

Montane temperate Kurils-Japanese-Korean endemic species is known in northern and middle Japan (until Shikoku), South Korea and South Kurils (Iturup Island), likely more widely distributed, at least to Kamchatka Peninsula in in the north. In Korea, Jeju-do, Chungcheongbuk-do, Chungchengnam-do, Gyeongsangnam-do, Gangwon-do, Jeollabuk-do and Jeollanam-do (Choi et al. 2017).

#### Specimens examined.

**Chungcheongnam-do**: Mt. Daedun, 36°08'02.9"N, 127°18'29.1"E, 343 m, 31 Mar 2009, *S.S. Choi 3405* (JNU); **Gangwon-do**: Mt. Seorak, 38°07'21.0"N, 128°27'27.7"E, 1649 m, 21 Sep 2009, *S.S. Choi 5174* (JNU), Mt. Seorak, 38°07'42.2"N, 128°26'21.6"E, 1011 m, 14 Oct 2010, *S.S. Choi 8607* (JNU), Mt. Seorak, 38°07'52.7"N, 128°26'11.2"E, 937 m, 14 Oct 2010, *S.S. Choi 8632* (JNU); **Gyeongsangnam-do**: Mt. Jiri, 35°19'20.6"N, 127°44'59.4"E, 1134 m, 14 Jun 2009, *S.S. Choi 3745* (JNU), Mt. Jiri, 35°20'01.7"N, 127°43'55.1"E, 1713 m, 3 Oct 2011, *S.S. Choi 111079* (JNU), Mt. Namdeogyu, 30 Oct 2008, *S.S. Choi 1119* (JNU); **Jeju-do**: Erimok valley, 33°21'59.6"N, 126°30'40.3"E, 1591 m, 6 Sep 2012, *S.S. Choi 120765* (JNU), Erimok valley, 33°21'59.6"N, 126°30'40.27"E, 1615 m, 6 Sep 2012, *S.S. Choi 120797* (JNU), Mt. Halla, 33°22'02.2"N, 126°33'05.9"E, 1563 m, 8 Aug 2010, *S.S. Choi 7737* (JNU), Mt. Halla, 33°21'42.1"N, 126°32'02.8"E, 1861 m, 21 Sep 2012, *S.S. Choi 120904* (JNU), Hyodon stream, 33°18'21.4"N, 126°33'38.5"E, 469 m, 7 Aug 2010, *S.S. Choi 7638* (JNU), Witse Oreum, 33°21'33.4", 126°30'54.2"E, 1668 m, 7 Sep 2012, *S.S. Choi 120847* (JNU); **Jeollabuk-do**: Mt. Deogyu, 22 Nov 2008, *S.S. Choi site 2-35* (JNU), Mt. Jiri, 35°19'25.0"N, 127°41'36.8"E, 1300 m, 7 Oct 2009, *S.S. Choi 6090* (JNU), Mt. Jiri, 35°19'50.1"N, 127°41'33.5"E, 1100 m, 21 May 2010, *S.S. Choi 7383* (JNU); **Jeollanam-do**: Mt. Dureun, 5 Feb 2009, *S.S. Choi 3064* (JNU), Mt. Jiri, 35°19'15.3"N, 127°31'50.0"E, 755 m, 19 Sep 2009, *S.S. Choi 5043* (JNU).

#### Comments.

This species was regarded as the variety within M. emarginata subsp. tubulosa by Kitagawa (1963); however, we agree with Stephani (1901) and treat it as a separate species because of the differences in DNA sequences between two taxa (Bakalin et al. 2019). *Marsupella apertifolia* differs from *M. tubulosa* in mostly rounded lobe apices (versus mostly acute), more or less equal lobes (versus distinctly unequal), non-biconcentric oil bodies (versus biconcentric), and constant absence of red or purple pigmentation.

### Marsupella koreana

Taxon classificationPlantaeJungermannialesGymnomitriaceae

Bakalin et Fedosov, Cryptogamie, Bryologie 40(7): 67, 2019

1CB69DF6-49CC-5E34-8DB5-AC6E375A768A

Figure 3

#### Type.

South Korea, Gyeongsangnam-do, Jiri Mts., National Park, 7.V.2015, Bakalin V. A. Kor-28-4-15 (***holotype***VBGI!, ***isotypes*** MW!, JNU!).

#### Description.

Plants in loose mats, more or less rigid, strongly distichous, brownish green to deep green, brownish greenish and yellowish brownish, also brown with rusty tint to brown purple, 500.0–1100.0 μm wide and 10.0–25.0 mm long. Rhizoids absent or very few, but common in geotropic stolons, colorless to grayish, obliquely to erect spreading. Stem rarely produces normal ventral branches, whereas commonly with ventral geotropic leafless stolons, almost always with 1–2 subfloral ventral or lateral innovations near gynoecia; stem cross section nearly rounded to slightly transversely elliptic, differentiated into strata, with outer layer cells 10.0–13.0 μm along margin, unequally thickened, but with thin and easily destroying external side, with moderate to small concave trigones; scleroderm well developed, in 2–3 layers, walls very thick, sometimes with visible median lamina, 7.0–10.0 μm in diameter, but with lumen only 3.0–6.0 μm in diameter, trigones moderate to large, concave; inner cells irregular in shape, 10.0–15.0 μm in diameter, walls thickened, trigones moderate, concave. Leaves distichously arranged, transversely to subtransversely inserted, obliquely spreading and subtransversely oriented, margins narrowly recurved in the both (dorsal and ventral) sides, narrowly canaliculate (looks conduplicate) with ‘keel’ slightly arched or nearly straight (in poorly developed phases), divided by gamma-shaped sinus into two strongly unequal gibbous lobes, lobe apices acute to obtuse. Cells in the midleaf mostly oblong, rarer subisodiametric, 7.0–20.0 × 7.0–13.0 μm, walls thickened, trigones large, triangle to convex, cuticle smooth, cells along lobe margin 5.0–10.0 μm, with unequally thickened walls, trigones small to moderate in size, concave, cuticle smooth; cells in the lobe middle 7.0–15.0 × 7.0–12.0 μm, with walls thickened to thin, trigones large and convex, sometimes confluent; oil-bodies (1–)2(–3) per midleaf cell, not biconcentric, spherical to oblong, ca. 5.0–7.5 × 5.0 μm. Dioicous. Androecia intercalary, with 2–3 pairs of bracts (but adjacent 1–2 pairs of ‘sterile’ leaves are similar with bracts that gives the impression of long androecia), spicate, bracts cupped to spoon-shaped, with recurved margin, suborbicular and lacerate when flattened in the slide, divided by **γ**-shaped sinus into two almost equal gibbous lobes, 750.0–875.0 × 825.0–1050.0 μm, 2–3-androus, antheridium stalk biseriate, 100.0–200.0 μm long, body nearly spherical ca. 100.0–120.0 μm in diameter. Perianth hidden within bracts or shortly exerted, onion-shaped, ca. 250.0 × 600.0 μm; perigynium well developed, 600.0–800.0 μm long (when archegonia fertilized), with two pairs of bracts, bracts sheathing perianth, with lobes incurved to perianth or very narrowly spreading. Elaters entirely bispiral, ca. 200.0 × 7.0–8.0 μm, with narrowed (sometimes even homogenous as in ‘*Plectocolea*-type’) ends. Spores brown, papillose, spherical, 10.0–11.0 μm in diameter.

**Figure 3.**
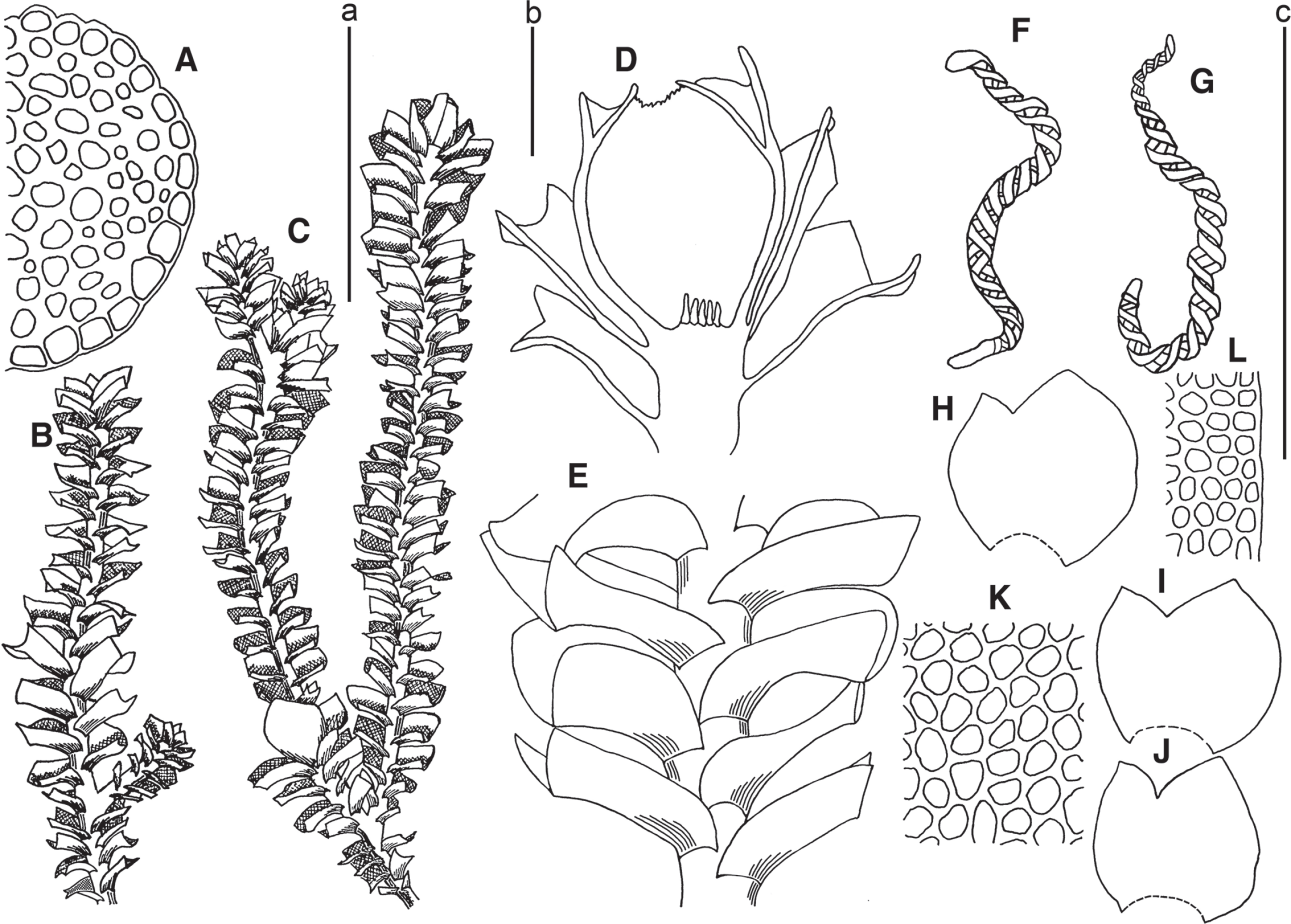
*Marsupella koreana* Bakalin et Fedosov **A** stem crops section (fragment) **B** male plant habit **C** female plant habit **D** gynoecium longitudinal section **E** shoot (fragment) **F, G** elaters **H–J** leaves **K** midleaf cells **L** cells along leaf margin. Scale bars: a 100 µm (**A, F, G, K, L**); b 1 mm (**B, C**); c 1 mm (**D, E, H–J**). All from *Choi 3511* (JNU).

#### Ecology.

Acidophilic to neutro-tolerant meso- to hygrophyte. The ecology of this species is somewhat similar to that of *M. pseudofunckii*. It occupies mesic, rarely moist, or dry substrates in open to partly shaded areas. Among the common associates in drier habitats, *Sphenolobus minutus*, in open subalpine stations, it sometimes grows together with *Gymnomitrion parvitextum*. In wetter habitats *M. koreana* may grow with *Marsupella tubulosa* and *Cephalozia otaruensis*.

#### Distribution.

Montane temperate species, known from only the southern part of the Korean Peninsula, but probably spreading northward. In Korea, Jeju-do, Chungchengnam-do, Gyeongsangnam-do, Jeollabuk-do and Jeollanam-do (Bakalin et al. 2019).

#### Specimens examined.

**Chungcheonnam-do**: Mt. Daedun, 36°08'02.9"N, 127°18'29.1"E, 343 m, 31 Mar 2009, *S.S. Choi 3407* (JNU); **Gyeongsangnam-do**: Mt. Gaya, 35°49'14.8"N, 128°07'27.5"E, 1313 m, 8 Sep 2009, *S.S. Choi* (JNU), Mt. Gaya, 35°47'30.1"N, 128°05'46.3"E, 521 m, 28 Apr 2009, *S.S. Choi 3511* (JNU), Mt. Gaya, 35°49'30.7"N, 128°07'07.9"E, 1350 m, 22 Jun 2010, *S.S. Choi 7402* (JNU), Mt. Jiri, 35°18'51.9"N, 127°44'22.1"E, 848 m, 13 Jun 2009, *S.S. Choi 3628* (JNU), Mt. Jiri, 35°19'20.6"N, 127°44'59.4"E, 1134 m, 14 Jun 2009, *S.S. Choi 3686* (JNU), Mt. Namdeogyu, 31 May 2008, *S.S. Choi site 1-2* (JNU), Mt. Namdeogyu, 35°45'53.5"N, 127°40'55.5"E, 1422 m, 11 Nov 2010, *S.S. Choi 8950* (JNU), **Jeju-do**: Mt. Halla, *S.S. Choi 111147* (JNU); **Jeollabuk-do**: Mt. Deogyu, 22 May 2008, *S.S. Choi 509* (JNU), Mt. Jeoksang, 35°57'24.3"N, 127°41'86.3"E, 724 m, 18 Mar 2009, *S.S. Choi 3417* (JNU), Mt. Jiri, 35°19'25.0"N, 127°41'36.8"E, 1300 m, 7 Oct 2009, *S.S. Choi 1005-1* (JNU), Mt. Jiri, 35°19'06.1"N, 127°31'47.5"E, 781 m, 20 Jun 2009, *S.S. Choi 4000* (JNU); **Jeollanam-do**: Mt. Dureun, 5 Feb 2009, *S.S. Choi 3058* (JNU), Mt. Jiri, 35°17'44.0"N, 127°31'59.0"E, 1421 m, 29 Apr 2009, *S.S. Choi 3527* (JNU).

#### Comments.

This distinctive species is one of the most common Korean *Marsupella* members, completely misidentified with a couple of other taxa (most frequently with *M. yakushimensis*, *M. apertifolia*, *M. pseudofunckii* and *M. tubulosa*). It belongs to the peculiar group of East Asian *Marsupella* taxa with ‘scapanioid’ appearance. Tentatively, we suggest that *M. koreana* occurs in mainland China (we were unable to check whether specimens identified as *M. pseudofunckii* from China (Gao and Wu 2007) are in fact *M. koreana*), as well as in Japan. We were unable to find this taxon in areas adjacent to the Korean Peninsula northward, in the Primorsky Territory of Russia. The main distinctions between the mentioned morphologically related taxa are presented in Table 1. *Marsupella koreana* is most morphologically similar to *M. patens* (N. Kitag.) Bakalin et Fedosov (the taxon not present in Korea proper and probably limited to the Japanese Archipelago) and *M. pseudofunckii*. The main distinctions of the former are in recurved leaf margins that are always flat or undulate in *M. patens* as well as in acute to obtuse (but in any way angular) lobe end, versus rounded in *M. patens*. The distinctions from *M. pseudofunckii* are the presence of narrowly recurved leaf margins in *M. koreana*, shoots not or slightly dilated to the perianth (versus distinctly dilated in *M. pseudofunckii*) and narrowly canaliculate, but not keeled-conduplicate (as in *M. pseudofunckii*) leaves.

**Table 1.** Distinction of *Marsupella koreana* from morphologically similar taxa.

Features	*M. pseudofunckii*	*M. apertifolia*	*M. yakushimensis*	*M. tubulosa*	*M. patens*	*M. koreana*
Lobes comparative size	unequal	more or less equal	more or less equal	subequal to slightly unequal	unequal	unequal
Leaf margin	Plane	recurved (at least in basal part)	plane to undulate	plane to indistinctly recurved near base	plane	recurved
Lobe apex	acute to obtuse	rounded	acute to obtuse	acute to obtuse	rounded	acute to obtuse
Cell wall in the midleaf	thin to slightly thickened	thin to slightly thickened	thick	thin to slightly thickened	thin to slightly thickened	thin to slightly thickened
Trigones in the midleaf	convex	convex	concave	convex to triangle	convex to triangle	convex
Leaf basal part	unistratose	unistratose or bistratose in large phases	bistratose	unistratose	unistratose	unistratose
Leaf shape	strongly conduplicate	concave-canaliculate to concave and nearly flattened	strongly conduplicate	canaliculate-concave to concave and canaliculate	canaliculate-concave	canaliculate
Purple shoot pigmentation	very rare	sometimes present	common in Japan, but rare in Korea	very common	common	common

### Marsupella pseudofunckii

Taxon classificationPlantaeJungermannialesGymnomitriaceae

S.Hatt., J. Hattori Bot. Lab. 4: 63, 1950

6B00F31B-9C40-563E-A634-29AAC6A4FAAE

Figure 4

#### Type.

Japan, Echime Prefectura, Omogo, 27 July 1940, *S. Hattori 5540* (***holotype*** TNS [174467!])

#### Description.

Plants in loose mats, more or less rigid, erect to ascending, strongly distichous, brownish green to deep green, rarely brown with rusty, purplish or reddish tint near apices, strongly dilated to the perianth, 500.0–600.0 μm wide in normally developed part, with common depauperate plants (in shady and dry habitats) starting from 150.0 μm wide, near perianth much wider and reaching 1100.0 μm, 10.0–25.0 mm long. Rhizoids virtually absent with exception of leafless geotropic stolons, where common, obliquely spreading, separated or united into unclear fascicles. Stem branching ventral and (more commonly) lateral, as subfloral innovations and ventral leafless geotropic stolons that sometimes transform into normal branches; stem cross section transversely elliptic, differentiated into strata, outer layer with walls unequally thickened (but external wall thin), 12.0–25.0 μm along margin, trigones moderate, concave, scleroderm cells 10.0–13.0 μm in diameter, with strongly thickened walls and moderate in size, concave trigones, gradually transformed to inner tissue, cells in inner part 10.0–18.0 μm in diameter, walls thickened to almost thin, trigones moderate, concave. Leaves transversely inserted, not or barely sheathing stem in base, obliquely spreading, transversely oriented, evidently keeled-conduplicate with plane margin, keel straight to slightly arched or suddenly turned downward near the end, leaves contiguous to distant, rarely enclosed one to another, 220.0–430.0 μm long and 300.0–600.0 μm wide, obliquely transversely elliptic, divided by **γ**-shaped sinus descending to 1/4–1/3 of leaf length into two strongly unequal gibbous lobes with acute to obtuse or very rarely rounded apes. Cells in the midleaf 10.0–18.0 × 7.0–15.0 μm, walls thin, trigones moderate in size to large, triangle to convex, cuticle smooth; cells along margin 5.0–8.0(–10.0) μm, thin to (more commonly) thickened, trigones small to large, mostly concave, cuticle smooth; cells in lobe middle 7.0–15.0 × 6.0–12.0 μm, walls thin to thick, trigones moderate to large, triangle to convex. Dioicous. Androecia intercalary, spicate, with 2–3 pairs of bracts, sequential generations divided by 10 and more pairs of sterile leaves, 1–3-androus, stalk biseriate, 125.0–220.0 μm long, body nearly spherical, ca. 150.0 μm in diameter; bracts cupped to spoon-shaped, commonly with recurved margin. Perianth hidden within bracts or barely exerted, couple-shaped, ca. 150.0 μm long and 400.0 μm wide, perigynium well developed, 500.0–700.0 μm long, with two pairs of bracts; bracts sheathing perigynium and above incurved inward to the perianth if archegonia were fertilized, or obliquely spreading, if archegonia not fertilized.

**Figure 4.**
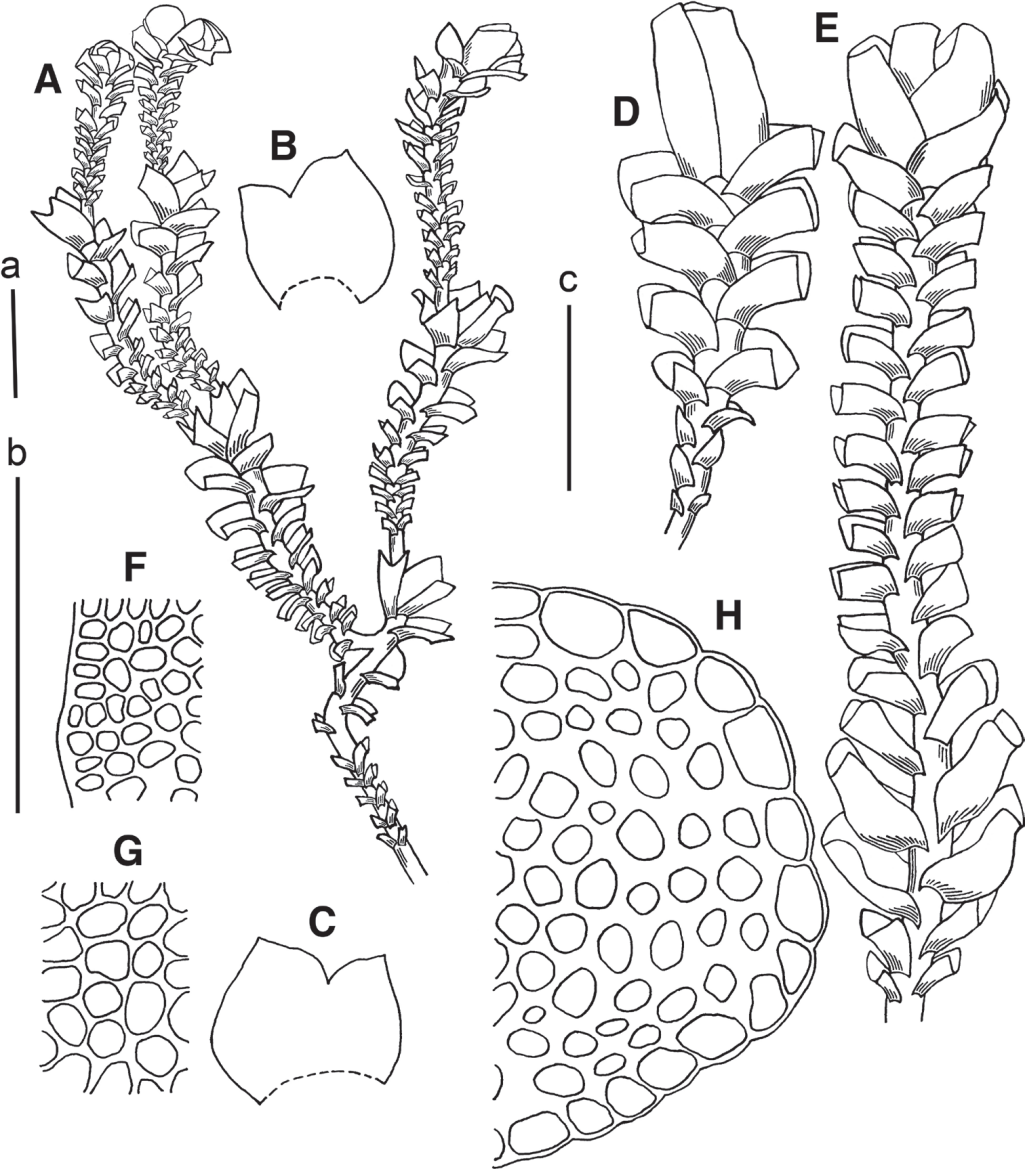
*Marsupella pseudofunckii* S.Hatt. **A** plant habit **B, C** leaves **D** female plant (fragment) **E** male plant (fragment) **F** cells along leaf margin **G** midleaf cells **H** stem cross section. Scale bars: a 1 mm (**A**); b 100 µm (**F–H**); c 1 mm (**B–E**). All from *Choi 545* (JNU).

#### Ecology.

Acidophilic meso- to hygrophyte taxon. The species occupies dry to moist cliffs, rarely wet rocks as well as stones near streams, in open to (more commonly) partly shaded places. It grows together with various *Scapania* and *Cephaloziella* or *Marsupella tubulosa* in wetter habitats. In dry to mesic and shady conditions, it is associated with *Tetralophozia filiformis*, *Bazzania ovifolia*, *Cylindrocolea recurvifolia*, *Diplophyllum taxifolium*, *D. albicans*, and *Syzygiella autumnalis*.

#### Distribution.

Temperate Montane East Asian species, aside from Korea known from China (Taiwan, Zheijiang (the report from the latter is based on Zhu et al. 1998)), Russian Far East (southern Sikhote-Alin only), and Japan. In Korea, Jeju-do, Chungchengnam-do, Gyeongbuk-do, Gyeongnam-do, Gangwon-do, and Jeollabuk-do (Kim and Hwang 1991; Yamada and Choe 1997). This species is here newly recorded from Gyeongsangbuk-do Province.

#### Specimens examined.

**Chungchengnam-do**: Mt. Daedun, 36°08'02.9"N, 127°18'29.1"E, 343 m, 31 Mar 2009, *S.S. Choi 3414* (JNU), Mt. Gyeryong, 36°21'06.9"N, 127°12'50.9"E, 290 m, 8 Jul 2009, *S.S. Choi 4083* (JNU); **Gangwon-do**: Mt. Seorak, 38°06'02.8"N, 128°23'43.1"E, 840 m, 28 Aug 2009, *S.S. Choi 4257* (JNU), Mt. Seorak, 38°06'35.7"N, 128°25'33.7"E, 1449 m, 21 Sep 2009, *S.S. Choi 5146* (JNU), Mt. Seorak, 38°07'55.5"N, 128°25'42.3"E, 805 m, 14 Oct 2010, *S.S. Choi 8655* (JNU); **Gyeongsangbuk-do**: Is. Ulleung, Mt. Mireuk, 26 Aug 2008, *S.S. Choi site5-191* (JNU), Seonginbong, 22 Aug 2008, *S.S. Choi site6-48* (JNU); **Gyeongsangnam-do**: Mt. Jiri, 35°20'02.4"N, 127°43'54.2"E, 1720 m, 15 Jun 2009, *S.S. Choi 3772* (JNU); **Jeju-do**: Mt. Halla, 33°21'45.3"N, 126°32'08.9"E, 1916 m, 8 Aug 2010, *S.S. Choi 7757* (JNU); **Jeollabuk-do**: Mt. Deogyu, 22 May 2008, *S.S. Choi 545* (JNU), Mt. Jeoksang, 35°57'24.3"N, 127°41'86.3"E, 724 m, 18 Mar 2009, *S.S. Choi 3416* (JNU), Mt. Naejang, 35°29'16.2"N, 126°53'46.3"E, 250 m, 16 Mar 2009, *S.S. Choi 3482* (JNU); **Jeollanam-do**: Mt. Dureun, 5 Feb 2009, *S.S. Choi 3057* (JNU), Mt. Jiri, 35°17'44.0"N, 127°31'59.0"E, 1421 m, 29 Apr 2009, *S.S. Choi 3537* (JNU).

#### Comments.

This is a distinctive species belonging to the group of *Marsupella* with conduplicate leaves. It may be mistaken for *M. koreana* (distinguishing characters given under that species) or (less probably) for *M. yakushimensis*. The latter and *M. pseudofunckii* share conduplicate leaves with not revolute margins, although being different in: 1) lobes are subequal in *M. yakushimensis*, but strongly unequal in *M. pseudofunckii*, 2) leaf margin of *M. yakushimensis* is commonly undulate and lobe ends turned away of the stem, whereas leaf margin in *M. pseudofunckii* is always plane and not reflexed away from the stem, 3) shoots of *M. pseudofunckii* are strongly dilated to the perianth, versus almost stable shoot width in *M. yakushimensis*, 4) the width of sterile shoots of *M. pseudofunckii* generally less than 0.6 mm, but in *M. yakushimensis* it commonly is more than 1.5 mm.

### Marsupella tubulosa

Taxon classificationPlantaeJungermannialesGymnomitriaceae

Steph., Bull. Herb. Boiss. 5: 99, 1897

C8952539-9EB9-56C9-97DA-E7DF0B253ECF

Figure 5

Marsupella emarginata var. tubulosa (Steph.) N.Kitag., Mem. Coll. Sci. Kyoto Imp. Univ., Ser. B, Biol. 27: 77, 1960.

#### Type.

Japan, Unzen, 5 Mar 1895, *Faurie 15385* (***lectotype*** (designated here) G [15042/00061032!])

#### Description.

Plants merely rigid, forming loose patches, deep green-brown, purple-brown, purple-green, or rarely greenish (actually plants extracted from the patch yellowish brownish in general, but with purple-rusty coloration in apices and upper parts of insolated leaves that gives expression of purple-brown color of patch), or yellowish greenish, pale brownish with purple tint in apical parts, rarer brownish greenish without purple or rusty pigmentation; 0.6–1.2 mm wide (the largest lax plants up to 1.3–1.5 mm) and 5.0–15.0 mm long. Rhizoids nearly absent or few, colorless. Stem brownish, not branched or branched as ventral leafless stolons (rarely becoming to normal branch) or more commonly as subfloral ventral or lateral innovations; transversely elliptic in cross section, 160.0–180.0 μm high and 200.0–250.0 μm wide (depauperate shoots omitted), differentiated into strata; hyaloderm with external wall thin, radial walls thin to unequally thickened (becoming thicker inward), inner wall thick, 15.0–20.0 μm along margin; scleroderm in (1–)2 rows of cells, cells thick-walled, but not so strongly as in *M. apertifolia*; inner cells thin to slightly thickened, trigones moderate in size, concave, 12.0–18.0 μm in diameter. Leaves contiguous to distant, sometimes ‘enclosed’ one to another, concave to almost flattened in upper half, transversely inserted, evidently or very loosely sheathing stem in the base, obliquely (rarely erect) spreading, subtransversely to obliquely oriented, suborbicular to widely ovate, margin flat to loosely recurved near base (especially in the leaves sheathing the stem), divided into two unequal (rarely subequal) lobes by widely V- to **γ**-shaped sinus descending to 1/7–1/5(1/4) of leaf length, lobe apex couple-shaped, obtuse to acute. Cells in the midleaf shortly oblong, 10.0–20.0(–25.0) × 8.0–16.0(–18.0) μm, walls thin to slightly thickened, trigones large to (rarer) moderate in size, convex to bulging, cuticle smooth; cells along leaf margin 6.0–12.0 μm, walls thin to slightly (to strongly and unequally) thickened in tangential walls, trigones moderate to large, concave to slightly convex, tangentially sometimes confluent; cells in lobe middle oblong, 15.0–22.0 × 8.0–13.0 μm, walls thin to slightly thickened, trigones moderate to large, convex to triangle. Oil-bodies in the midleaf cells 2(–3) per cell, biconcentric (at least (30–)70%), finely to normally papillose, spherical, ca. 5.0 μm in diameter to oblong, 7.5–12.5 × 5.0–7.5 μm. Dioicous. Androecia intercalary, with 1–3 or 3–5 pairs of bracts, different generations divided by 4–8 pairs of sterile leaves, spicate, 3–5-androus, stalk biseriate, 75.0–100.0 μm long, body ellipsoidal, ca. 140.0 × 115.0 μm; bracts spoon-shaped, nearly subquadrate when flattened, with deflexed to erect-spreading lobes. Perianth onion-shaped, hidden within bracts, ca. 300.0 × 500.0 μm; perigynium 500.0–700.0 μm long, with (1–)2 pairs of bracts; bracts sheathing perianth near base and erect spreading in upper 1/3–1/2. Capsule elliptic, outer layer cells rectangular, 27.0–50.0 × 20.0–25 μm, with 2(–4) nodular thickenings in vertical walls and 1(–3) thickenings in horizontal wall; inner cells elongate and flexuous, 45.0–63.0 × 7.0–10.0 μm, with 7–10 sometimes bifurcate semicircular bands. Elaters (2–)3-spiral, 150.0–180.0 × 7.0–8.0 μm. Spores brown, papillose, spherical, 10.0–12.0 μm in diameter.

**Figure 5.**
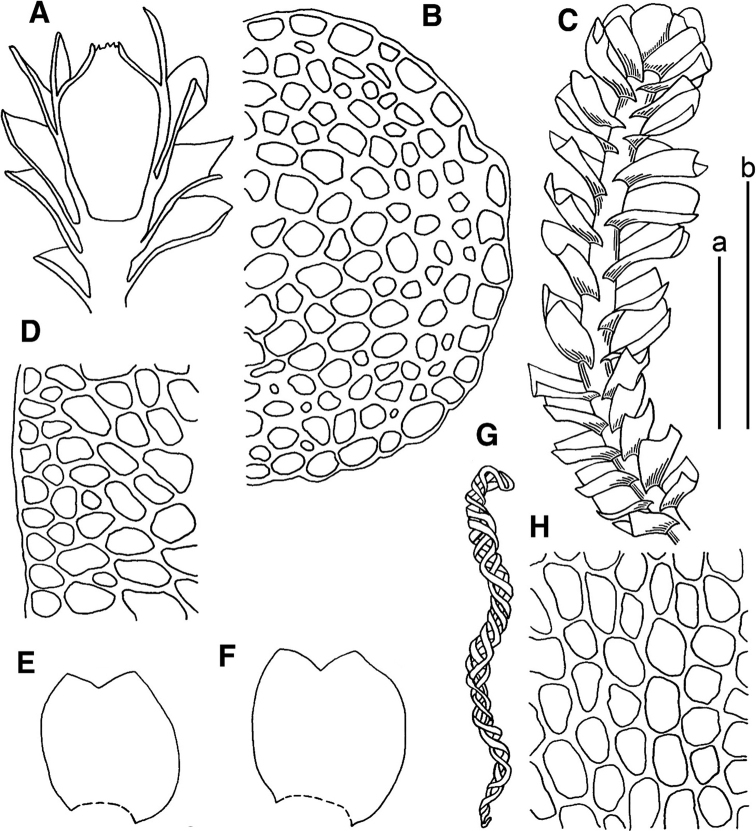
*Marsupella tubulosa* Steph. **A** gynoecium longitudinal section **B** stem cross section (fragment) **C** plant habit, dorsal view **D** leaf margin cells **E, F** leaves **G** elater, **H** midleaf cells. Scale bars: 1 mm (**A, C, E, F**); b 100 µm (**B, D, G, H**). All from *Choi 3732* (JNU).

#### Ecology.

Acidophilic meso- to hygrophyte. The species occupies sandy soils and mineral substrates, over wet to moist, and sometimes mesic cliffs, being most common along streams near running water. In drier habitats, it is commonly associated with *Odontoschisma pseudogrossiverrucosum*, *Cheilolejeunea obtusifolia*, and rarely with *Microlejeunea punctiformis*, *Cephaloziella* spp., *Gymnomitrion faurianum*. In wetter habitats *M. tubulosa* sometimes grows intermixed with *Solenostoma minutissimum*, *Lophocolea horikowana*, *Marsupella pseudofunckii*, and *M. koreana*.

#### Distribution.

The distribution of the species is confined to insular and peninsular areas in Amphi-Pacific Boreal and Temperate Eastern Asia, while the records provided by Bakalin, (2010), Cherdantseva and Gambaryan (1986), Gambaryan (1992, 2001), Konstantinova et al. (2002) for the Russian Far East continental mainland are likely incorrect. This species is strikingly characterized by biconcentric oil bodies – an uncommon feature in *Marsupella* that has never been reported from continental Asia or from North America. This suggests the reports of the species in Schuster (1974) for North America and Schljakov (1981) for Russian Asia are incorrect. We suggest the ‘true’ *M. tubulosa* (the type is from Honshu) occurs only in Japan, Kurils, Kamchatka and the Korean Peninsula, as well as probably in China (Anhui, Taiwan), from where, unfortunately, oil bodies were not studied. All specimens collected in the continental mainland of the Russian Far East and checked alive had non-biconcentric oil bodies, whereas specimens from peninsular and insular parts of the Far East possess biconcentric oil bodies. The species was recorded for nearly all provinces of the Korean Peninsula (Jeju-do, Gyeongsangnam-do, Gyeongsangbuk-do, Chungcheongnam-do, Chungcheongbuk-do, Gyeonggi-do, Gangwon-do, Pyeonganbuk-do, Hamgyeongnam-do, Hamgyeongbuk-do: Yamada and Choe 1997; Kim and Hwang 1991) and was confirmed for most of the provinces in the southern part of the peninsula.

#### Specimens examined.

**Chungcheongnam-do**: Mt. Daedun, 36°08'02.9"N, 127°18'29.1"E, 343 m, 31 Mar 2009, *S.S. Choi 3399* (JNU), Mt. Gyeryong, 36°21'06.9"N, 127°12'50.9"E, 290 m, 8 Jul 2009, *S.S. Choi 4098* (JNU); **Gangwon-do**: Mt. Seorak, 38°06'02.8"N, 128°23'43.1"E, 840 m, 28 Aug 2009, *S.S. Choi 4258* (JNU), Mt. Seorak, 38°06'39.7"N, 128°24'56.2"E, 1347 m, 21 Sep 2009, *S.S. Choi 5095* (JNU), Mt. Seorak, 38°07'21.0"N, 128°27'27.7"E, 1649 m, 21 Sep 2009, *S.S. Choi 5175* (JNU); **Gyeongsangbuk-do**: Is. Ulleung, Seonginbong, 37°29'39.5"N, 130°52'35.4"E, 845 m, 20 Oct 2010, *S.S. Choi 8712* (JNU), 37°29'53.9"N, 130°52'01.0"E, 977 m, 20 Oct 2010, *S.S. Choi 8744* (JNU); **Gyeongsangnam-do**: Mt, Gaya, 35°48'53.9"N, 128°07'21.9"E, 1116 m, 8 Sep 2009, *S.S. Choi 4361* (JNU), 35°49'14.8"N, 128°07'27.5"E, 1313 m, 8 Sep 2009, *S.S. Choi 4376* (JNU), Mt. Jiri, 35°18'51.9"N, 127°44'22.1"E, 848 m, 13 Jun 2009, *S.S. Choi 3641* (JNU), 35°19'50.7"N, 127°44'08.1"E, 1540 m, 14 Jun 2009, *S.S. Choi 3732* (JNU); **Jeju-do**: Bolre Oreum, 33°21'20.8"N, 126°28'10.2"E, 1145 m, 5 Sep 2012, *S.S. Choi 120721* (JNU), Erimok valley, 33°21'59.6"N, 126°30'40.25"E, 1613 m, 6 Sep 2012, *S.S. Choi 20795* (JNU), Mt. Halla, 33°21'43.1"N, 126°32'21.9"E, 1835 m, 8 Aug 2010, *S.S. Choi 7745* (JNU), Musu stream, 33°25'08.0"N, 126°26'56.4"E, 495 m, 28 Oct 2010, *S.S. Choi 8827* (JNU), Suak valley, 33°20'14.0"N, 126°36'37.8"E, 523 m, 29 Oct 2010, *S.S. Choi 8879* (JNU); **Jeollabuk-do**: Mt. Deogyu, 30 Jun 2008, *S.S. Choi 887* (JNU), Mt. Jiri, 35°19'06.1"N, 127°31'47.5"E, 781 m, 20 Jun 2009, *S.S. Choi 3993* (JNU); **Jeollanam-do**: Mt. Dureun, 5 Feb 2009, *S.S. Choi 3061* (JNU), Mt. Jogyeo, 34°59'51.6"N, 127°16'51.7"E, 288 m, 7 Dec 2010, *S.S. Choi 9094* (JNU).

#### Comments.

This species may be confused with at least three other species: *Marsupella koreana*, *M. apertifolia*, and *M. emarginata*, though the last is not known from the Korean Peninsula. The distinctions between the former two are mentioned under those species. The species differs from *M. emarginata* (with which *M. tubulosa* was probably confused in parts outside of oceanic/suboceanic Eastern Asia) in biconcentric oil bodies (versus oil bodies without the central eye) and commonly obliquely oriented leaves that are not closely sheathing the stem in the base (versus transversely oriented leaves with leaves closely sheathing the stem near base). The latter feature is surely quantitative and variation in leaf orientation occurs within the two species.

### Marsupella vermiformis

Taxon classificationPlantaeJungermannialesGymnomitriaceae

(R.M.Schust.) Bakalin et Fedosov, Cryptogamie, Bryologie 40(7): 70, 2019

E879EF68-18C3-5BB8-8AF0-FF4CBAA33819

Figures 6 , 7I–K

Basionym. Marsupella stoloniformis subsp. vermiformis R.M.Schust., J. Hattori Bot. Lab. 80: 72, 1996

#### Type.

Malaysia. North Borneo: Mt. Kinabalu National Park, S. slope, around Paca Cave, 2985 m, Mizutani, 2788 (not seen).

#### Description.

Plants strongly vermicular, forming loose patches, brown to blackish brown, without red or purple pigmentation, orbicular in cross section, 100.0–140.0 μm in diameter, 3.0–6.0 mm long, freely ventrally branched, from leafless brownish to whitish in color densely ventrally branched rhizome. Rhizoids virtually absent, to solitary, colorless, obliquely spreading, short (less than 100.0 μm long). Stem 100.0–170.0 μm in diameter, orbicular in cross section, outer layer cells with external wall thin to obscurely thickened, tangential walls subequally thickened, trigones small, concave, walls brown in color, 6.0–10.0 μm in diameter, inner cells with walls unequally thickened, walls colorless, trigones moderate in size, concave. Leaves appressed to the stem (commonly lacerate into two segments when try to detach), transversely inserted and oriented, not decurrent, widely triangular, 65.0–110.0 μm long and 90.0–175.0 μm wide, divided by V-shaped sinus descending to 2/5–1/2 of leaf length into two subequal triangle lobes with acute apices. Cells in the midleaf 5.0–10.0 × 5.0–8.0 μm, walls moderately thickened, trigones small, concave; cuticle smooth; oil-bodies 1–2 per cell, spherical, 2.0–3.0 μm in diameter. Dioicous. Pants suddenly dilated to the perianth, to form club-shaped structure, perianth completely hidden within bracts, nearly conical, 75.0–100.0 μm long and 200.0–230.0 μm wide, smooth, perigynium 120.0–150.0 μm long, with one pair of bracts; bracts nearly orbicular to orbicular-triangular in shape, ca. 250.0 × 250.0 μm, covering perianth and then occlude one with another.

**Figure 6.**
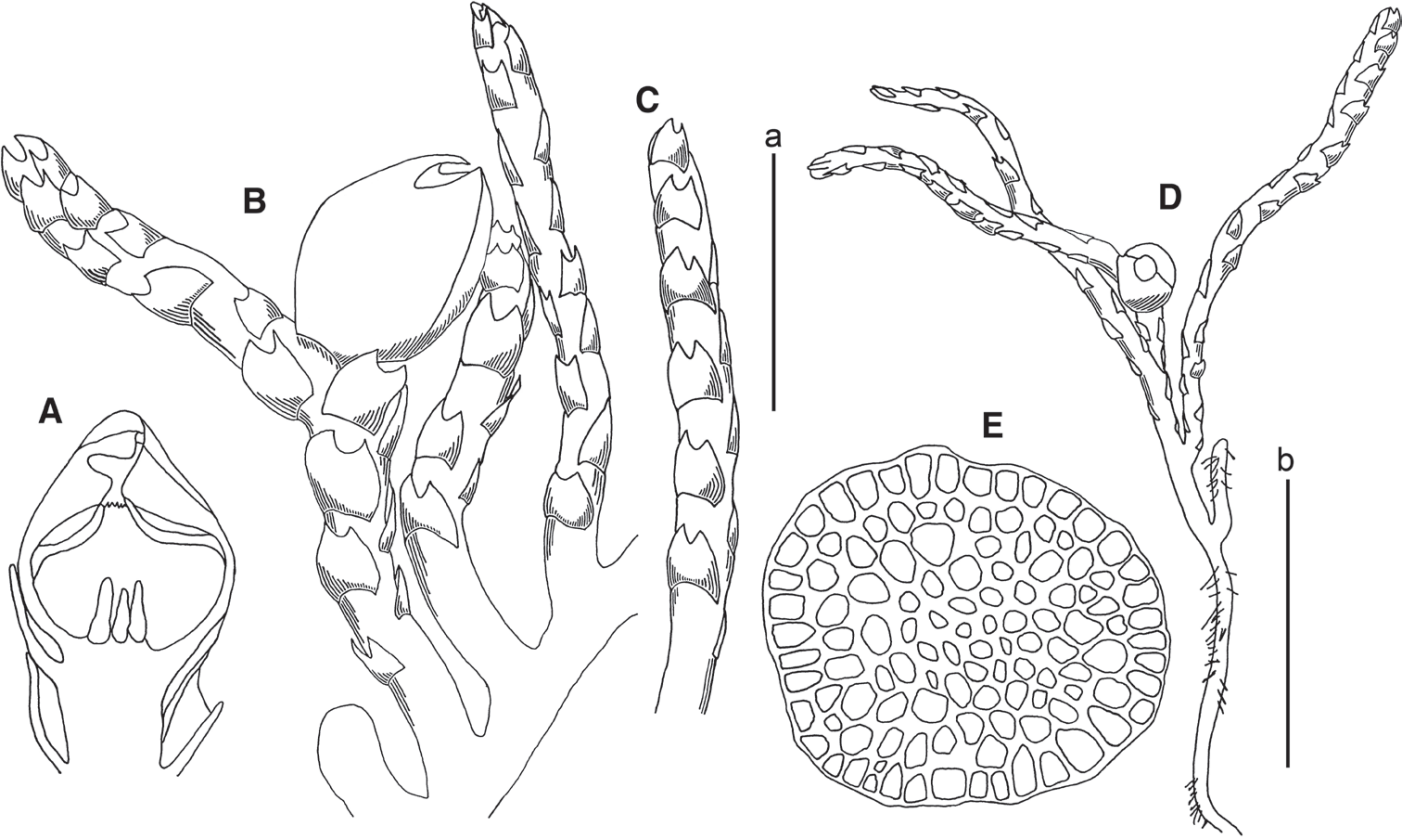
*Marsupella vermiformis* (R.M.Schust.) Bakalin et Fedosov **A** gynoecium longitudinal section **B, C** plant habit, fragments **D** plant habit **E** stem cross section. Scale bars: a 500 µm (**A–C**); a 1 mm(**D**); b 100 µm (**E**). All from *Choi 120911* (JNU).

**Figure 7.**
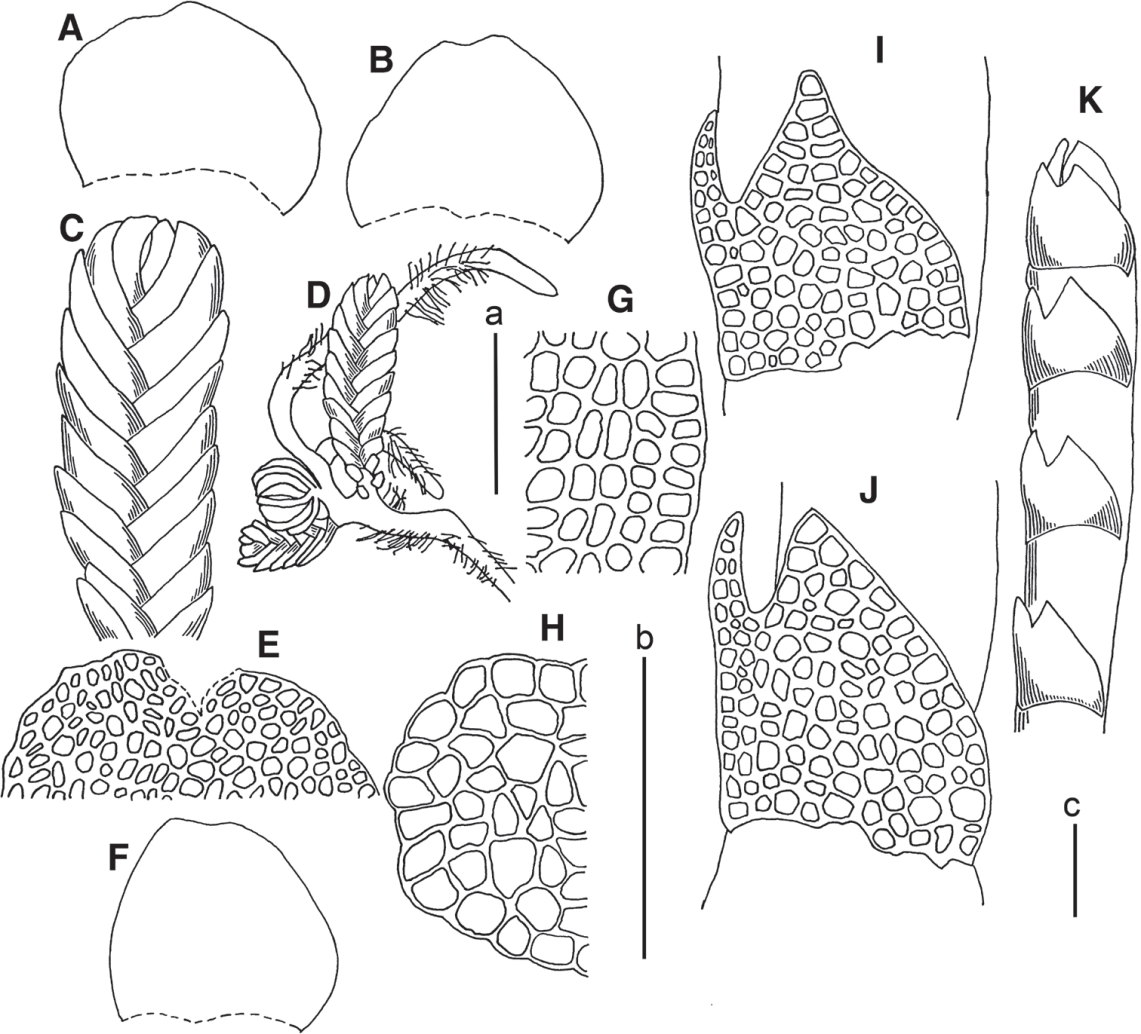
*Gymnomitrion noguchianum* S.Hatt. **A, B, F** leaves **C** plant habit (fragment) **D** plant habit **E** lacerate leaf apex **G** cells along leaf margin **H** stem cross section (fragment). All from *Choi 120826* (JNU); *Marsupella vermiformis* (R.M.Schust.) Bakalin et Fedosov **I, J** leaves **K** plant habit (fragment). All from *Choi 120911* (JNU). Scale bars: a 250 µm (**A–C, F**); a 500 µm (**D**); b 100 µm (**E, G–J**); c 100 µm (**K**).

#### Ecology.

Acidophilic meso-xerophyte. In Korea, it occurs on dry well-exposed rocks in large block gravelly barrens in the crater rim of Halla Mt. *Marsupella vermiformis* formed pure patches or a slight admixture of *Gymnomitrion faurianum* or dwarf form of *Marsupella tubulosa*.

#### Distribution.

Strongly disjunct rare taxon ranging the area from southernmost Korea (Jeju-do) across China, to Malaysia and Papuasia (Bakalin et al. 2019). The species belong to the oligotypic section Stolonicaulon (N.Kitag.) Vaňa, which it shares with rare South and South-East Asian *M. stoloniformis* N.Kitag.

#### Specimens examined.

**Jeju-do**: Mt. Halla, 33°21'42.1"N, 126°32'02.8"E, 1861 m, 21 Sep 2012, *S.S. Choi 120911, 120897* (JNU, VBGI).

#### Comment.

The very distinctive species, superficially quite similar to *Gymnomitrion pacificum* Grolle due to vermicular shoots and never spreading, but closely appressed leaves, forming in female branches, a club-like structure. It is clearly different from *G. pacificum* in having much smaller leaves, with normally developed cells along the margin and presence of distinct perianth. The species may be mistaken for dwarf forms of arctic-alpine sub-circumpolar amphi-oceanic *Marsupella boeckii* (Austin) Lindb. ex Kaal. and, possible, European (amphi-Atlantic) – British Columbian (cf. Paton 1999) *Marsupella stableri* Spruce. However, besides distinct gaps in distribution this species differs: 1) the never spreading leaves in *M. vermiformis* versus at least slightly spreading in perianthous shoots in *M. boeckii* and obliquely spreading to squarrose in *M. stableri*, 2) shoot width not exceeding 140.0–170.0 μm, versus 200.0–500.0 μm in *M. boeckii*, and 100–400 μm in *M. stableri*, 3) small cells with brown colored, thickened tangential walls in stem cross section in *M. vermiformis*, versus large and hyaline cells in *M. boeckii* and *M. stableri*), 4) small leaves that are wider than long, reaching at maximum 175.0 μm wide and 110 μm long, versus 200–300 μm wide in *M. boeckii* and distinctly longer than wide (up to 300 × 200 μm) in *M. stableri*, 5) very small leaf cells, 5.0–10.0 × 5.0–8.0 μm in *M. vermiformis*, versus 12.0–20.0 × 12.0–20.0 μm in *M. boeckii* and 10–16 μm in diameter in *M. stableri*. The distinction from *M. stoloniformis* (hardly possible in Korea) as well as the phylogenetic position of both is discussed by Bakalin et al. (2019).

### Marsupella yakushimensis

Taxon classificationPlantaeJungermannialesGymnomitriaceae

(Horik.) S.Hatt., Bull. Tokyo Sci. Mus. 11: 80, 1944

9EC59667-16E0-5730-8D44-CF56ABF9F58A

Figure 8

Basionym. Sphenolobus yakushimensis Horik., J. Sci. Hiroshima Univ., Ser. B, Div. 2, Bot. 2: 156, 1934

#### Type.

Japan. Kagoshima Pref., Yakushima Island, Horikawa, 11895 (not seen).

#### Description.

Plants in loose patches, deep green-brown, yellow-brown, yellowish brownish, rarely with purple tint, (1.0)1.5–2.1 mm wide and 15.0–50.0 mm long, rigid. Rhizoids nearly absent to very sparse, colorless, obliquely spreading, however common in basal part of ventral branches and leafless stolons. Stem easily laterally and ventrally branched giving start to normal branches or geotropic leafless stolons; stem transversely elliptic in cross section 210.0–240.0 μm high and 250.0–320.0 μm wide, distinctly differentiated into strata, hyaloderm cell walls moderately thickened (but external wall thin), with small concave trigones, 17.0–25.0 μm along margin, scleroderm cells with very thick walls and visible median lamina, 12.0–17.0 μm in diameter, but with lumen disappearing or only 2.0–6.0 μm in diameter, inner cells with moderately thickened walls and moderate in size, concave trigones, 10.0–15.0 μm in diameter. Leaves strongly conduplicate and distichously arranged that gives ‘scapanioid’ appearance, contiguous to imbricate, as a rule enclosed one to another, obliquely spreading and transversely oriented, when flattened subquadrate, rectangular or obovate to suborbicular (mostly wider than long, but sometimes longer than wide), bistratose in lower 1/5–1/6 of the leaf length, 675.0–1250.0 μm long and 800.0–1500.0 μm wide, commonly dorsally secund, divided by **γ**-shaped sinus descending to 1/4–2/5 of leaf length into two equal to subequal lobes (either ventral or dorsal may be smaller), lobes gibbous, with obtuse to acute or rarely rounded apex. Cells in the midleaf subisodiametric to (mostly) oblong, 12.0–25.0 × 7.0–20.0 μm, strongly thick-walled, with moderate to small, concave trigones, cuticle smooth; cells along leaf margin 7.0–10.0 μm, thick-walled (but with much thinner external wall), with moderate in size, concave trigones; cells in lobe middle 10.0–17.0 × 8.0–15.0 μm, thick-walled, with small to moderate in size, concave trigones, cuticle smooth. Dioicous. Androecia intercalary, with 2–3 pairs of bracts, spoon-shaped, with revolute margin and commonly deflexed lobe ends. Perianth (only unfertilized were found) hidden within bracts, onion-shaped, perigynium the same length with perianth or slightly longer, with 2 pairs of bracts and 1–3 lateral and ventral subfloral innovations.

**Figure 8.**
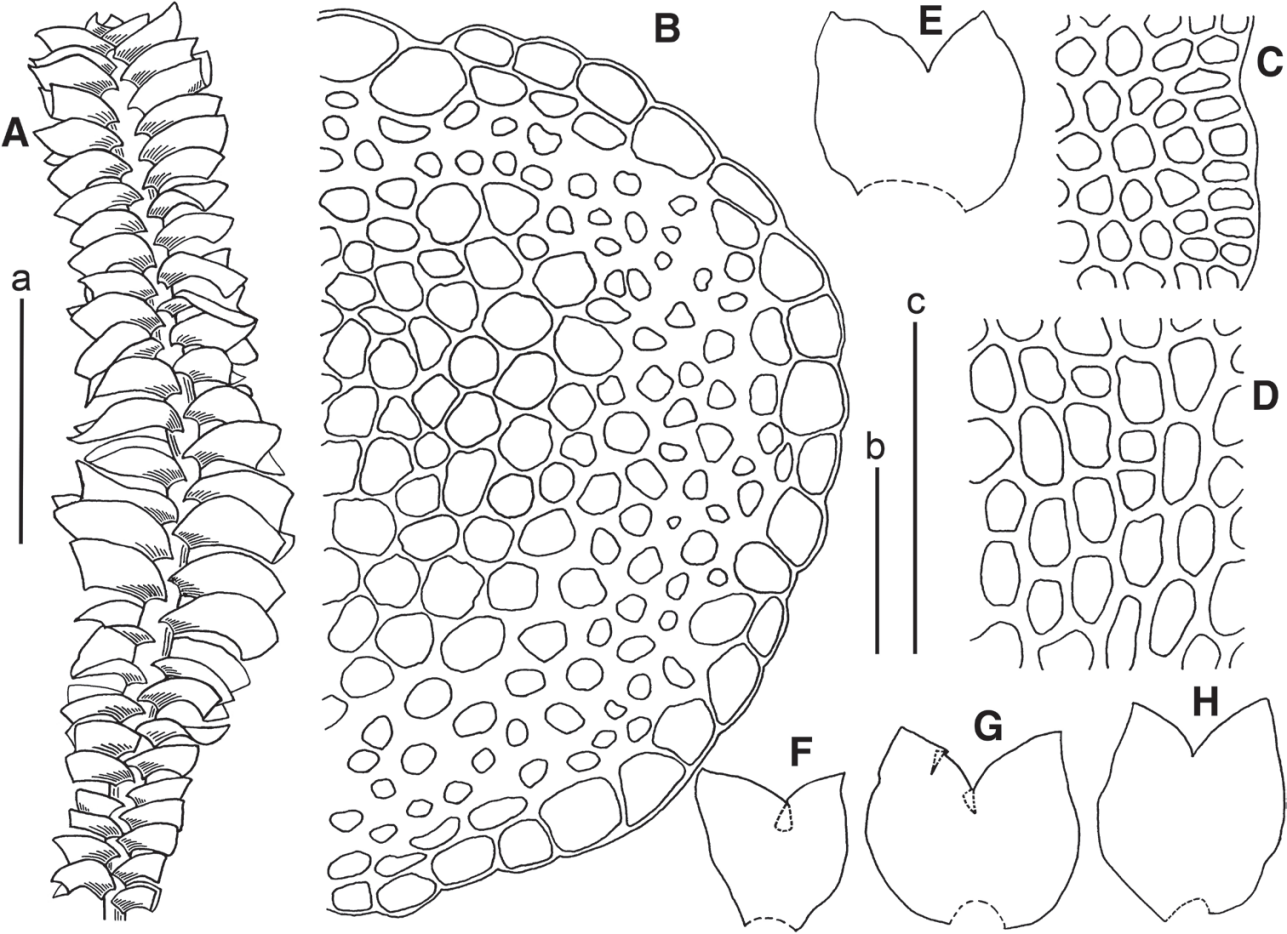
*Marsupella yakushimensis* (Horik.) S.Hatt. **A** plant, dorsal view **B** stem cross section (fragment) **C** cells along leaf margin **D** midleaf cells **E–H** leaves. Scale bars: a 2 mm (**A**); b 1 mm (**E–H**); c 100 µm (**B–D**). All from *Choi-1067* (JNU).

#### Ecology.

Acidophilic hygro- to hydrophyte. The species occurs on wet cliffs at a distance from watercourses or on stones washed with sluggishly running water in partly shaded habitats in the middle elevation of mountains covered with evergreen to deciduous broadleaved forests. Commonly, the species forms pure patches or rarer, associated with *Scapania undulata*.

#### Distribution.

South temperate to subtropical Montane East Asian endemic species known in China (Anhui, Fujian, Guangdong, Jiangxi, Zhejiang), the southern part of the Korean peninsula (the report by Kim and Hwang (1991) for North Korea is doubtful) and the southern half of Japan. The species was reported from Gyeongsangnam-do, Gangwon-do, Gyeongbuk-do (Kim and Hwang 1991; Yamada and Choe 1997) and here added to Jeollabuk-do and Jeju-do. The specimen included in the phylogenetic tree in Bakalin et al. (2019) under the name *Marsupella alata* S.Hatt. et N.Kitag. (Republic of Korea, Seorak Mt., 11.V.2011, Bakalin, Kor-6-28a-11, VBGI) was re-studied and found as the dwarf modification of *M. yakushimensis*. Although the distinctive differences between cited specimen and another accession of *M. yakushimensis* may suspect more robust than infraspecific differences that should be considered in future studies of the genus in East Asia.

#### Specimens examined.

**Gangwon-do**: Mt. Seorak, 38°09'34.3"N, 128°28'10.5"E, 631 m, 11 Oct 2010, *S.S. Choi 8347* (JNU); **Gyeongsangnam-do**: Mt. Jiri, 35°19'58.3"N, 127°44'27.5"E, 1327 m, 4 Oct 2011, *S.S. Choi 111125* (JNU); **Jeollabuk-do**: Mt. Jiri, 35°19'25.0"N, 127°41'36.8"E, 1300 m, 7 Oct 2009, *S.S. Choi 6083* (JNU), Mt. Jiri, *S.S. Choi 1067* (JNU); **Jeju-do**: Seogwipo-si, 33°18'30"N, 126°30'30"E, 600–800 m alt. 13 May 2015, *V.A. Bakalin s.n.* (VBGI).

#### Comments.

This large and beautiful species is a rarity within the Korean flora and is known only from a few localities. Unlike Japanese populations, the Korean populations acquire purple to red pigmentation as an exception. The main characteristic of the species includes nearly equal lobes that do not have recurved margins, but commonly undulate and/or turned antically. Another characteristic feature is the absence of a distinctly sheathing leaf base. Dwarf plants of *M. yakushimensis* may be mistaken for *M. koreana*, and the distinctions are given under the latter. This species is regularly observed with androecia and rarely with archegonia. Androecious and gynoecious plants were intermixed within two specimens; however, we were unable to observe fertilized (in at least two descendant generations) and fully developed perianth. Whether this is the norm or not is not clear.

### Gymnomitrion

Taxon classificationPlantaeJungermannialesGymnomitriaceae

Corda, Naturalientausch 12: 651, 1829.

457D0FE5-C619-5FB4-8C19-1DD5B55C9257

#### Description.

Plants worm-shaped to ribbon-like, with densely imbricate leaves or similar to *Marsupella* and with loosely spreading leaves, rigid to soft, whitish to brownish, brown, rusty, and blackish brown, without red or purple pigmentation. There are two kinds of phenotypes: *Gymnomitrion* in the old sense and the former genus *Apomarsupella* R.M. Schust. nested within Gymnomitrion (cf. Shaw et al. 2015). The first phenotype, with imbricate leaves and stems creeping to ascending, subclavate, from rhizomatous base, dorsiventrally compressed, commonly immersed to the substrate and incrusted by soil particles. The second phenotype comprises plants with spreading leaves with shoots dorsiventrally not compressed, as well as having a not evident rhizomatous base. In both ‘phenotypes’ rhizoids are common in the rhizomatous shoot base and geotropic stolons, but rare in leafy parts of the shoot, soft, colorless to grayish or rarely and solitarily deep purple. Stem not evidently different in strata, rather it is monomorphic (outer layer cells slightly larger), cells with unequally thickened walls, and well-developed trigones. Leaves lobed to unlobed or shallowly emarginate, with plane or narrowly recurved margin. Leaf cells pachydermous, commonly with the rim of discolored cells. Dioicous (taxa known in Korea). Androecia intercalary, (1–)2(–3)-androus, stalk biseriate. Perigynium and perianth virtually absent or strongly reduced. Elaters bispiral.

#### Comment.

This treatment follows the recent emendations *e.g*. transfer to *Gymnomitrion* of the taxa *Marsupella commutata* (Limpr.) Bernet and *Apomarsupella revoluta* (cf. Shaw et al. 2015). These transfers made *Marsupella* more monomorphic in the series of features, but resulted in greater polymorphism in vegetative characters of *Gymnomitrion*, which now includes many taxa of ‘marsupelloid’ habit, although in reproductive characters is characterized by an absence, or strong reduction of, perianth and perigynium.

### Key to *Gymnomitrion* taxa recorded in Korea

**Table:** 

1	Leaves entire to emarginate, never distinctly bilobed	***G. noguchianum***
–	Leaves distinctly bilobed	**2**
2	Plants from huge rhizomatous base, leaved part clavate, leaves densely imbricate, leaf margin plane and discolored	**3**
–	Plants lacking huge rhizomatous base, not or scarcely clavate, leaves loosely spreading, margin not discolored, revolute (at least in base)	**5**
3	Branches vermicular, brownish to nearly brown (sometimes pure green in Europe, but such forms were never seen in Eastern Asia), cells along leaf margin nearly thick-walled, with large and convex trigones, never erose [not confirmed for Korea]	[***G. concinnatum***]
–	Branches dorsiventrally compressed, whitish to grayish, rarely yellowish brownish, cells along leaf margin thin-walled	**4**
4	Leaves with acute lobes, cells along lobe margin thin-walled, with trigones distinct, mostly with sharply verruculose cuticle (cf. Bakalin, 2016, Figure 3: Q-X), leaf margin persistent	***G. faurianum***
–	Leaves with rounded lobe apices, cells along lobe margin thin-walled (easily destroyed in older parts), trigones virtually absent, cuticle smooth [not confirmed for Korea]	[***G. corallioides***]
5	Black to black-brown plants, trigones in the midleaf concave, in apical part of the leaf triangle and never quadrate, leaf margin always strongly revolute, leaf cuticle coarsely verrucose [not confirmed for Korea]	[***G. revolutum***]
–	Plants yellowish, yellow-brown, and yellowish green to somewhat whitish green, rarely blackish brown, trigones in the midleaf bulging, in lobe apex commonly quadrate and giving appearance of chessboard, margin recurved to somewhat plane in upper part of the leaf, leaf cuticle verrucose to smooth	**6**
6	Plants yellowish green to whitish green, somewhat dorsiventrally compressed, leaves distichously arranged, leaf margin crenulate due to projecting cell walls, commonly with hemispherical papillae	***G. parvitextum***
–	Plants blackish brown, not compressed dorsiventrally, leaves sheathing the stem, not distichous, leaf margin entire, cuticle smooth to finely verrucose, never with hemispherical papillae	***G. commutatum***

### Gymnomitrion commutatum

Taxon classificationPlantaeJungermannialesGymnomitriaceae

(Limpr.) Schiffn., Magyar Bot. Lapok 13: 304, 1914 [1915].

3A9F4B2E-8449-5E5F-8628-33136B6B9342

Marsupella commutata (Limpr.) Bernet, Cat. Hép. Suisse: 29, 1888
Basionym. Sarcocyphos commutatus Limpr., Jahresber. Schles. Ges. Vaterl. Cult. 57: 314, 1879 [1880].

#### Type.

Austria. Tirol, Montefuner Tal, 2300 m, 1868, leg. Jack, (***lectotype*** BP (not seen)).

#### Description.

Plants in loose patches, rigid, slightly glistening when dry, hardly soaking, blackish brown, without red or purple pigmentation, 450.0–700.0 μm wide and 5.0–15.0 mm long. Rhizoids nearly absent, with the exception of ventral stolons, where common (sometimes dense), colorless or with admixture of solitary deep purple. Stem brownish to whitish (commonly whitish in geotropic stolons), branching lateral or ventral, rather common as subfloral innovations, also as ventral stolons with scale-like leaves; transversely elliptic in cross section, 120.0–140.0 μm high and 130.0–150.0 μm wide, composed by rather uniform cells, outer layer cells 12.0–20.0 μm along margin, slightly larger than inner cells, with brownish and unequally thickened walls and large (sometimes confluent) concave trigones; inner cells 10.0–18.0 μm, walls unequally thickened, colorless, trigones large, triangular to convex. Leaves imbricate, enclosed one to another, concave-canaliculate to concave and spoon-shaped, transversely inserted, sheathing the stem, loosely obliquely spreading and transversely oriented, sometimes secund dorsally, elliptic to loosely widely ovate or obovate or nearly rectangular, 320.0–500.0 μm long and 300.0–450.0 μm wide, margin recurved to plane in upper part of the leaf, divided by V-shaped sinus, with commonly recurved basal part of the sinus, descending to 1/4–1/3 of leaf length, into two equal to subequal lobes, lobes triangular to gibbous with obtuse to acute, rectangular or even rounded apex. Cells in midleaf 8.0–20.0(–23.0) × 8.0–17.0 μm, walls thin, trigones large, bulging, cuticle smooth to finely verrucose; cells along margin 6.0–11.0 μm, with thin to thickened walls, trigones large, bulging or convex, sometimes confluent, in robust phases external wall protruding, the margin then crenulate, cuticle smooth to verrucose; cells in lobe middle 7.0–13.0 × 7.0–12.0 μm, thin-walled, with large bulging or quadrate and confluent trigones (gives expression of chessboard), cuticle smooth to verrucose.

#### Ecology.

Acidophilic meso-xerophyte, the species occupies more or less dry substrata in exposed to (rarely) partly shaded areas. In the study area was intermixed with *Gymnomitrion noguchianum*.

#### Distribution.

*Gymnomitrion commutatum* was described based on plants from Austria. Váňa et al. (2010: 20) gave its distribution as “Northern Europe, Middle Europe, Southwestern Europe, Southeastern Europe, Siberia, Russian Far East, China, Eastern Asia, Indian Subcontinent, Malesia, Subarctic America, Western Canada, Northwestern USA”. Many of the Asian records may belong to other taxa, for instance, *Gymnomitrion parvitextum*, discussed below (Mamontov et al. 2018). In turn *G. commutatum* may possess disjunctive arctic-alpine distribution. Within Pacific Asia *G. commutatum* is known from the Russian Far East, Japan, and likely may be found in China. This species is only found in the Halla-san crater rim. It is noteworthy that this species is known from Jeju-do, whereas the morphologically similar *G. parvitextum* (see below) is not found there, but is quite common in other provinces of Korea.

#### Specimens examined.

**Jeju-do**: Mt. Halla, 33°21'51.0"N, 126°31'42.9"E, 1814 m, 7 Sep 2012, *S.S. Choi 120826* (JNU), 33°21'42.1"N, 126°32'02.8"E, 1861 m, 21 Sep 2012, *S.S. Choi 120924* (JNU), Mansedongsan valley, 33°21'59.6"N, 126°30'40.3"E, 1591 m, 6 Sep 2012, *S.S. Choi 120834* (JNU).

#### Comment.

The species is very similar to *G. parvitextum*, and the distinctions between the two taxa are described below.

### Gymnomitrion faurianum

Taxon classificationPlantaeJungermannialesGymnomitriaceae

(Steph.) Horik., Acta Phytotaxonomica et Geobotanica 13: 212. 1943

13977514-3C3C-5E74-BBA8-B09A54130268

Figure 9

Basionym. Acolea fauriana Steph. Species Hepaticarum 2: 8. 1901

#### Type.

Japan. “Tidesan” 29 August, 1898, Faurie 212 (***lectotype*** (designated here) G [00067200/15025!])

#### Description.

Plants in loose mats, more or less soft, leaved part of shoot distinctly clavate, from rhizomatous base, dorsiventrally compressed, whitish to whitish green and pale brownish in general aspect, due to discolored leaf margins that gives expression of white pigmentation, although middle part of leaves maybe greenish to brownish green, 250.0–800.0 μm wide (large plants to 1000.0 μm wide) and 5.0–15.0 mm long. Rhizoids virtually absent to sparse in leaved shoots, but rather common in rhizomatous base and geotropic leafless stolons, soft, colorless, in indistinct obliquely spreading fascicles, rarely with admixture of solitary purple in color and rigid rhizoids. Stem in leaved part branched as subfloral innovations, while in rhizomatous base freely and variously branched, leafless geotropic stolons infrequent, originated mostly near base of leaved part of shoot; cross section not differentiated into distinct layers, nearly orbicular to slightly transversely elliptic, 140.0–160.0 μm high and 170.0–190.0 μm long; outer cells slightly larger than inner, 20.0–30.0 μm along margin with walls thick (but external wall thinner), trigones moderate in size, concave; inner cells irregular in shape, with flexuous thickened walls, 15.0–23.0 μm in diameter, trigones moderate in size, concave. Leaves densely imbricate, enclosed one to another, obliquely spreading, transversely oriented, not sheathing in the base, ovate to obliquely ovate and widely triangular in shape, 350.0–700.0 μm long and 400.0–650.0 μm wide, divided by V-shaped sinus descending to 1/7 (smaller plants from drier habitats) – 1/6–1/5 of leaf length into two equal to subequal triangular to loosely gibbous lobes with acute to obtuse apes and distinctly crenulate margins. Cells in the midleaf subisodiametric to rectangular and irregularly oblong, 17.0–30.0 × 17.0–23.0 μm, thin-walled, with moderate to large, convex trigones, cuticle smooth; cells along margin 12.0–18.0 μm, thick- to merely thin-walled, with thinner external wall, discolored in 1–5 cells rows almost to the leaf base, with trigones moderate to small in size, concave, cuticle sharply verruculose; cells in lobe middle 15.0–30.0 × 12.0–(18.0)20.0 μm, walls thin to thickened, trigones varying from moderate to small and from concave to bulging (if trigones become bulging cell walls become thinner), cuticle verruculose to smooth. Dioicous. Androecia intercalary. Perianth and perigynium absent, bracts similar to leaves, but larger, more deeply divided (up 15–1/4 of the length), with somewhat diverging and spreading, rarely erose-dentate lobes.

**Figure 9.**
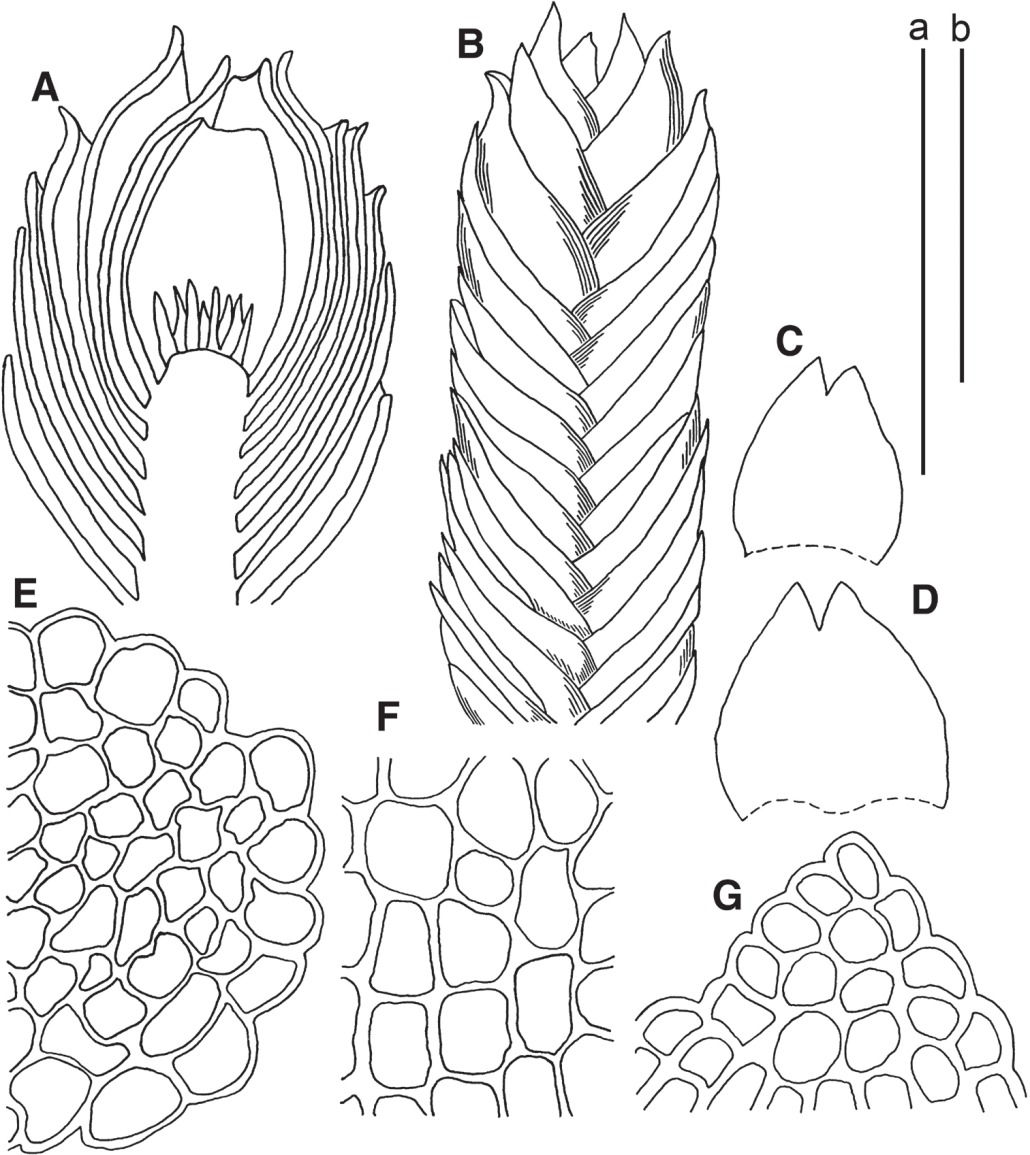
*Gymnomitrion faurianum* (Steph.) Horik. **A** gynoecium longitudinal section **B** plant habit **C, D** leaves **E** stem cross section (fragment) **F** midleaf cells **G** cells in leaf lobe apex. Scale bars: a 1 mm (**A–D**); b 100 µm (**E–G**).

#### Ecology.

Acidophilic mesophyte. The species occupies mesic to moist (rarely wet) cliffs in open places, and rarely occurs in partly shaded habitats, producing thinner forms with not densely appressed leaves. In drier habitats, it is commonly associated with *Gymnomitrion parvitextum*, dwarf forms of *Sphenolobus saxicola*, saxicolous modifications of *Anastrophyllum michauxii*, in more wet habitats it may be mixed with *Marsupella pseudofunckii* and *Protolophozia debiliformis*.

#### Distribution.

East Asian oro-boreal species widely distributed in northern to middle Japan (although a rarity as far as Yakushima Island), abundant in southern Kurils. Within the Asian mainland, known from Sikhote-Alin mountain system, stretching to South Korea until Jeju-do. Likely to be found in North-East China (at least in Changbai Mountain). Previous reports of *Gymnomitrion concinnatum* (Lightf.) Corda from Yanggang-do and Pyeongannam-do (Kim and Hwang 1991) likely belong to this taxon.

#### Specimens examined.

**Gangwon-do**: Mt. Seorak, 38°06'35.7"N, 128°25'33.7"E, 1449 m, 21 Sep 2009, *S.S. Choi 5148, 5148-2* (JNU), Mt. Seorak, 38°07'09.4"N, 128°27'54.8"E, 1710 m, 21 Sep 2009, *S.S. Choi 5206* (JNU), Mt. Seorak, 38°08'02.8"N, 128°28'03.6"E, 908 m, 12 Oct 2010, *S.S. Choi 8378, 8381, 8384* (JNU), Mt. Seorak, 38°06'36.3"N, 128°25'33.6"E, 1452 m, 13 Oct 2010, *S.S. Choi 8502* (JNU), Mt. Seorak, 38°07'08.9"N, 128°27'55.4"E, 1718 m, 13 Oct 2010, *S.S. Choi 8557* (JNU); **Jeju-do**: Mt. Halla, 33°21'45.3"N, 126° 32'08.9"E, 1916 m, 8 Aug 2010, *S.S. Choi 7759* (JNU), Mt. Halla, 33°21'45.3"N, 126°32'08.9"E, 1916 m, 8 Aug 2010, *S.S. Choi 7765a* (JNU), Mt. Halla, *S.S. Choi 120372* (JNU), Mt. Halla, 33°21'51.0"N, 126°31'42.9"E, 1814 m, 7 Sep 2012, *S.S. Choi 120827* (JNU), Mt. Halla, 33°21'42.1"N, 126°32'02.8"E, 1861 m, 21 Sep 2012, *S.S. Choi 120890, 120894, 120907, 120909, 120924, 120925, 120936, 120937, 120938* (JNU).

#### Comment.

The whitish plant coloration, distichous leaf arrangement, and distinctly bilobed leaves easily help in recognizing *G. faurianum*. The difference from *G. corallioides* is the presence of persistent cells along the leaf margin and verrucose cuticle in the leaf lobes, as was noted in the key. Moreover, we consider the occurrence of *G. corallioides* in Korea unlikely. Another problem is the differentiation of *G. faurianum* from *G. concinnatum*, which shares the papillose cuticle in the leaf apex and stout cell walls in the leaf margins. However, *G. faurianum* differs from *G. concinnatum* in strongly distichous leaf arrangement, dorsiventrally compressed shoots and whitish coloration (although in Europe *G. concinnatum* may be sometimes whitish green and pure green, such modifications were never observed in East Asia) – the features rather resembling *G. corallioides*, which were the reasons for the misidentifications of *G. faurianum* for both *G. concinnatum* and *G. corallioides*, as discussed by Bakalin (2016).

### Gymnomitrion noguchianum

Taxon classificationPlantaeJungermannialesGymnomitriaceae

S.Hatt., J. Jap. Bot. 27: 55, 1952

D4329C52-D5A8-5FE3-9DFF-F5EA1BD913F6

Figure 7A–H

#### Type.

Japan. Tottori Prefecture, Daisen Mt., 1400 m, on volcanic rocks, 8 August 1947, A. Noguchi s.n. (***holotype***: NICH [13067!]).

#### Description.

Plants in loose mats, more or less soft, leaved part of shoots distinctly clavate from rhizomatous base, dorsiventrally compressed, whitish to whitish green or almost completely white, immersed to soil and incrusted by soil particles, 250–350 μm wide and 500–700 μm long in leaved part, rhizomatous part ca. 3–5 mm long. Rhizoids common to (in rhizomatous base) dense, erect to obliquely spreading, separated or in unclear fascicles. Stem freely ventrally branched in the base of leaved part and variously and feely branched in rhizome; slightly transversely elliptic in cross section, ca. 100 μm high and 120 μm wide, without evident differentiation into layers, outer cells 12–15 μm along margin, with thick walls (become noticeable thinner to thin in ventral epidermis), with moderate in size, concave trigones, inner cells 7–13 μm in diameter, irregular in shape, with thickened walls and moderate to small in size concave trigones. Leaves densely imbricate, transversely inserted, not sheathing at base, transversely oriented, widely ovate-lingulate to widely triangular, cupped to spoon shaped, lacerate when flattened, 220–330 μm long and 270–500 μm wide, with rounded to emarginate apex and entire to crenulate margin. Cells in the midleaf subisodiametric (mostly quadrate) to oblong (mostly rectangular), 12–25 × 12–20(23) μm, walls thickened, trigones small to moderate in size, concave, cuticle smooth; cells along leaf margin 5–15 μm, mostly elongate perpendicularly to the margin, walls unequally thickened, trigones small to moderate, concave. Dioicous. Androecia intercalary, with cupped and loosely imbricate bracts. Perianth and perigynium absent, perichaetial area of the shoot distinctly wider than below.

#### Ecology.

Acidophilic meso-xerophyte. The species occupies dry to mesic fine soils in well-exposed places at higher altitudes. It is commonly associated with dwarf xeric forms of *Marsupella tubulosa*, *Gymnomitrion parvitextum*, and *Cephaloziella divaricata*.

#### Distribution.

Temperate Montane Eastern Asian endemic species with distribution confined to Japanese Honshu and Kushu as well as the southern tip of Korea (Jeju Island).

#### Specimens examined.

**Jeju-do**: Mt. Halla, 33°21'51.0"N, 126°31'42.9"E, 1814 m, 7 Sep 2012, *S.S. Choi 120809, 120812, 120826* (JNU).

#### Comment.

Due to the presence of entire to emarginate leaves, *Gymnomitrion noguchianum* is unlikely to be mistaken for other members of this genus. However, it may be mistaken for *Cryptocoleopsis imbricata*, but that species is not yet known from the Korean Peninsula, though it should be expected to occur there. Both taxa are similar in prostrate growth, occurring in well-exposed places, and entire imbricate leaves. However, two taxa may be easily separated by 1) leaf cell walls are thickened and smaller in *G. noguchianum*, versus larger (more than 20 μm wide) and thin-walled in *Cryptocoleopsis imbricata*, 2) total absence of brown pigmentation in *G. noguchianum* versus almost constant presence in *Cryptocoleopsis*; and 3) presence of calyptral perigynium in *Cryptocoleopsis* – the structure does not occur in *Gymnomitrion*.

### Gymnomitrion parvitextum

Taxon classificationPlantaeJungermannialesGymnomitriaceae

(Steph.) Mamontov, Konstant. et Potemkin, Nova Hedwigia 106(1–2): 88, 2018

13795C1F-E2EA-5879-A974-8BBAE1AA47DB

Figure 10

Basionym. Marsupella parvitexta Steph., Bulletin de l’Herbier Boissier, ser. 2, No. 2: 165, 1901.

#### Type.

Japan, Tosa, Mt. Tsutsujo, August 1898, Inoue n. 22 (***lectotype*** (designated here): G [9470/00067518!]).

#### Description.

Plants in loose patches, rigid, slightly glistening when dry, hardly soaking, yellowish, yellow-brown and yellowish greenish, without red or purple pigmentation, 450.0–1000.0 μm wide and 3.0–20.0 mm long. Rhizoids nearly absent, with the exception of ventral stolons, where common (sometimes dense), colorless or with admixture of solitary deep purple. Stem brownish to whitish (commonly whitish in geotropic stolons), branching lateral or ventral, rather common as subfloral innovations, also as ventral stolons with scale-like leaves; transversely elliptic in cross section, 125.0–175.0 μm high and 150.0–190.0 μm wide, composed by rather uniform cells, outer layer cells 12.0–20.0 μm along margin, slightly larger than inner cells, with brownish and unequally thickened walls and large (sometimes confluent) concave trigones; inner cells 10.0–18.0 μm, walls unequally thickened, colorless, trigones large, triangle to convex. Leaves distichously spreading, enclosed one to another, concave-canaliculate to concave and spoon-shaped, transversely inserted, barely or not sheathing the stem in the base, transversely oriented, sometimes secund dorsally, elliptic to loosely widely ovate or obovate or nearly rectangular, 360.0–670.0 μm long and 500.0–625.0 μm wide, margin recurved to plane in upper part of the leaf, divided by V-shaped sinus, with commonly recurved basal part of the sinus, descending to 1/4–1/3 of leaf length, into two equal to subequal lobes, lobes triangular to gibbous with obtuse to acute, rectangular or even rounded apex. Cells in the midleaf 8.0–20.0(–23.0) × 8.0–17.0 μm, walls thin, trigones large, bulging, cuticle smooth; cells along margin 6.0–11.0 μm, with thin to thickened walls, trigones large, bulging or convex, sometimes confluent, in robust phases protrudent in external wall that gives expression of crenulate margin, cuticle smooth to verrucose; cells in lobe middle 7.0–13.0 × 7.0–12.0 μm, thin-walled, with large bulging or quadrate and confluent trigones (gives expression of chessboard), cuticle smooth to with hemispherical papillae. Dioicous. Androecia intercalary, with 2–4 pairs of bracts (adjacent pairs of leaves somewhat similar in shape with bracts that may be misinterpreted as bracts), spicate, 1(–2)-androus, stalk biseriate, ca. 100.0 μm long, body nearly spherical ca. 130.0–140.0 μm in diameter; bracts spoon-shaped, with more widely than in leaves recurved margin, widely ovate-trapezoidal when flattened. Perianth entirely absent; perigynium absent or very low (up 100.0 μm long); bracts similar to leaves, but longer; commonly with 1–2 subfloral innovations becoming into normal branch and fertilized soon again or forming flagelliform brown colored branch (in drier habitats).

**Figure 10.**
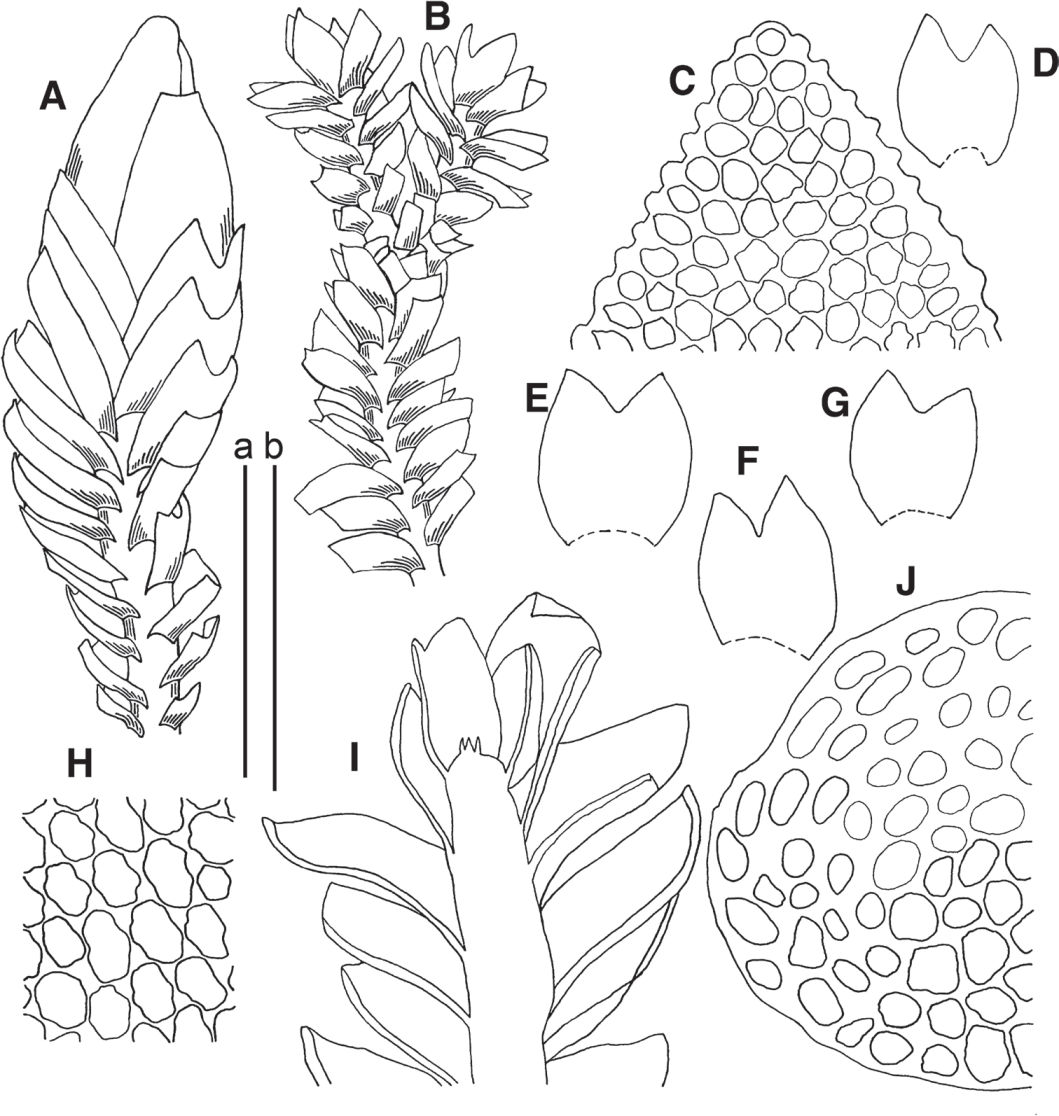
*Gymnomitrion parvitextum* (Steph.) Mamontov, Konstant. et Potemkin **A** female plant (fragment) **B** male plant (fragment) **C** leaf lobe apex **D–G** leaves **H** midleaf cells **I** gynoecium longitudinal section **J** stem cross section (fragment). Scale bars: a 1 mm (**A, B, D–G**); a 500 µm (**I**); b 100 µm (**C, H, J**). All from *Choi 8475* (JNU).

#### Ecology.

Acidophilic meso-xerophyte. The species occupies more or less dry substrata in exposed to (rarely) partly shaded habitats. It is commonly intermixed with *Marsupella tubulosa*, *M. koreana*, *M. pseudofunckii*, *Gymnomiotrion faurianum*, *Herbertus dicranus*, *Anastrophyllum assimile*, or (as exotic variant) with *Scapania ampliata*.

#### Distribution.

East Asian oro-boreal taxon, known in areas adjacent to the Korea from the Russian Far East, Japan, and likely should be found in China. It was recorded in Korea from Gyeonggi-do, Gyeongsangnam-do, Hamgyeongnam-do (Kim and Hwang 1991; Yamada and Choe 1997) under the name *Marsupella commutata*. It is also reported from Gangwon-do.

#### Specimens examined.

**Gangwon-do**: Mt. Seorak, 38°08'02.8"N, 128°28'03.6"E, 908 m, 12 Oct 2010, *S.S. Choi 8383* (JNU), Mt. Seorak, 38°07'34.9"N, 128°27'16.0"E, 1487 m, 12 Oct 2010, *S.S. Choi 8475* (JNU); **Gyeongsangnam-do**: Mt. Jiri, 35°19'20.6"N, 127°44'59.4"E, 1134 m, 14 Jun 2009, *S.S. Choi 3703* (JNU), 35°20'10.6"N, 127°43'42.0"E, 1820 m, 15 Jun 2009, *S.S. Choi 3816* (JNU), Mt. Namdeogyu, 35°45'53.5"N, 127°40'55.5"E, 1422 m, 11 Nov 2010, *S.S. Choi 8954* (JNU).

#### Comment.

Easily recognizable species in most cases, although confused several times with dwarf plants of *Marsupella tubulosa* from which it differs in commonly present subquadrate to quadrate trigones in leaf lobe cells, almost constantly recurved leaf margin, and in the absence of perianth. *Gymnomitrion parvitextum* is morphologically similar to *G. alpinum*. The two taxa are distinct in the presence of subquadrate trigones in leaf lobe cells in *G. parvitextum* (absent in *G. alpinum*), recurved margin (versus margin plane), shortly or barely decurrent leaf base (versus long decurent leaf base on both sides in *G. alpinum*) and inability to develop red or purple pigmentation (versus such coloration common in *G. alpinum*).

The bigger problem is the delimitation of *Gymnomitrion parvitextum* from morphologically similar and ‘semicryptic’ (Mamontov et al. 2018) *G. commutatum* (Limpr.) Schiffn., disjunct, mostly northern hemiarctic species. *Gymnomitrion parvitextum* differs from *G. commutatum* in the following features: 1) pale yellowish to brownish and even yellowish-greenish coloration, but never blackish brown and rusty-brown coloration so common in *G. commutatum*, 2) leaves semi-distichously arranged versus leaves subimbricate, 3) leaves in the vast majority of cases with widely recurved margins, versus margin recurved narrowly and commonly near the leaf base only.

### Excluded or doubtful taxa

#### Marsupella sphacelata

Taxon classificationPlantaeJungermannialesGymnomitriaceae

(Giesecke ex Lindenb.) Dumort., Recueil Observ. Jungerm.: 24, 1835.

4E453231-C812-5686-AC11-BC30439E9BDB

##### Remarks.

The taxon was recorded for North Korea (Gangwon-do, Hamgyeongnam-do, cf. Kim and Hwang 1991), but was not found in specimens examined. We suggest that all reports regarding the occurrence of this species in Korea and adjacent regions are erroneous and probably belong to *M. apertifolia* to which the species are similar in coloration and rounded leaf lobes, but differ in leaf margin characteristics, leaf sinus deepness, and stem cross section features, all of which are included in the identification key. In general, *M. sphacelata* is a rarity in East Asia and probably absent southward of 53°N (southern part of Kamchatka Peninsula). The records for Honshu Island in Japan are doubtful due to reported red pigmentation (Kitagawa 1963) which is not known for this species from other parts of its range (cf. Schuster 1974).

#### Gymnomitrion concinnatum

Taxon classificationPlantaeJungermannialesGymnomitriaceae

(Lightf.) Corda, Gen. Hepat.: 651, 1829

EBB5CDB1-4595-53AD-A165-6DF606924649

##### Remarks.

All reports of this taxon belong to *G. faurianum*, as discussed under the latter.

#### Gymnomitrion corallioides

Taxon classificationPlantaeJungermannialesGymnomitriaceae

Nees, Naturgesch. Eur. Leberm. 1: 118, 1833.

4F0CD00D-99CD-5177-A2D0-608B4681B72C

##### Remarks.

This taxon was recorded for Jeju-do (Yamada and Choe 1997) and identified many times in unpublished collections; however, all so-named specimens checked by us are characterized by acute lobes with persistent cells with moderate size trigones along the leaf margin. These features are characteristics of *Gymnommitrion faurianum*. Everywhere southward of 48°N. in East Asia *G. faurianum* was commonly misidentified as *G. corallioides* (Bakalin 2016).

#### Gymnomitrion revolutum

Taxon classificationPlantaeJungermannialesGymnomitriaceae

(Nees) H.Philib., Rev. Bryol. 17: 34, 1890.

98AB5A04-283B-5BA9-A3F3-63C22C7AE34C

Apomarsupella revoluta (Nees) R.M.Schust., J. Hattori Bot. Lab. 80: 82, 1996; M. revoluta (Nees) Trevis., Rendiconti Ist. Lomb. Sci. Lett. 7: 783, 1874.

##### Remarks.

This species was recorded on the Korean Peninsula from North Korea (Yanggang-do, cf. Kim and Hwang 1991) but the vouchers were unavailable for the present study. The species resembles *G. parvitextum*, and the main differences are included in the key. The occurrence of *G. revolutum* in the Korean Peninsula is somewhat probable. In adjacent countries, it was recorded in China, in areas remotely located from the Korean Peninsula (Xizang, Taiwan) and in Japan.

## Supplementary Material

XML Treatment for Gymnomitriaceae

XML Treatment for Marsupella

XML Treatment for Marsupella apertifolia

XML Treatment for Marsupella koreana

XML Treatment for Marsupella pseudofunckii

XML Treatment for Marsupella tubulosa

XML Treatment for Marsupella vermiformis

XML Treatment for Marsupella yakushimensis

XML Treatment for Gymnomitrion

XML Treatment for Gymnomitrion commutatum

XML Treatment for Gymnomitrion faurianum

XML Treatment for Gymnomitrion noguchianum

XML Treatment for Gymnomitrion parvitextum

XML Treatment for Marsupella sphacelata

XML Treatment for Gymnomitrion concinnatum

XML Treatment for Gymnomitrion corallioides

XML Treatment for Gymnomitrion revolutum

## Footnotes

* The nomenclature of taxa mentioned in the ‘Ecology’ section follows Söderström et al. (2016).

## References

[B1] Bakalin VA (2010) The distribution of Bryophytes in the Russian Far East. Part. 1. Hepatics. Publishing company of Far Eastern University, Vladivostok, 175 pp.

[B2] Bakalin VA (2016) Does Gymnomitrion corallioides Nees (Hepaticae) occur in Temperate East Asia? Botanica Pacifica 5(1): 53–61. 10.17581/bp.2016.05106

[B3] Bakalin VA Fedosov VE Fedorova AV Nguyen VS (2019) Integrative taxonomic revision of Marsupella (Gymnomitriaceae, Hepaticae) reveals neglected diversity in Pacific Asia. Cryptogamie. Bryologie 40(7): 59–85. 10.5252/cryptogamie-bryologie2019v40a7

[B4] Cherdantseva VY Gambaryan SK (1986) Bryophytes. Flora i rastitil’nost’ Bol’shekhekhcirskogo zapovednica. Dal’nauka, Vladivostok, 79–101.

[B5] Choi SS Bakalin VA Park SJ Ra EH (2017) Liverworts and Hornworts Flora of Korea, Volume 2 (Scapaniaceae–Dendrocerotaceae). National Institute of Biological Resources, Ministry of Environment, Korea, Incehon, 156 pp.

[B6] Gambaryan SK (1992) Anthocerotae and Hepaticae of Southern Primorye. Dal’nauka, Vladivostok, 175 pp.

[B7] Gambaryan SK (2001) Liverworts of the Sikhote-Alin Reserve (Primorsky Territory). Arctoa 10(1): 31–42. 10.15298/arctoa.10.04

[B8] Gao C Wu YH (2007) Flora Bryophytorum Sinicorum. Vol. 10. Jungermanniales (Lophoziaceae–Neotrichocoleaceae). Science Press, Beijing, 464 pp.

[B9] Kim YH Hwang HJ (1991) Korean Spore Plant 8 (Hepaticae). Publishing House of Science on Encyclopodia, Pyongyang, 223 pp.

[B10] Kitagawa N (1963) A revision of the family Marsupellaceae of Japan. The Journal of the Hattori Botanical Laboratory 26: 76–118.

[B11] Konstantinova NA Bakalin VA Potemkin AD Ignatov MS (2002) Hepatic flora of Upper Bureya River (Russian Far East). Arctoa 11(1): 393–398. 10.15298/arctoa.11.25

[B12] Mamontov YS Konstantinova NA Vilnet AA Potemkin AD Sofronova EV Gamova NS (2018) On resurrection of Marsupella parvitexta Steph. (Gymnomitriaceae, Marchantiophyta) as a semi cryptic species of the genus Gymnomitrion. Nova Hedwigia 106(1–2): 81–101. 10.1127/nova_hedwigia/2017/0466

[B13] Mamontov YS Vilnet AA Konstantinova NA Bakalin VA (2019) Two new species of Gymnomitriaceae (Marchantiophyta) in the North Pacific. Botanica Pacifica 8(1): 67–80. 10.17581/bp.2019.08113

[B14] Paton JA (1999) The liverwort flora of the British Isles. Harley Books, Colchester, 626 pp.

[B15] Piippo S (1990) Annotated catalogue of Chinese Hepaticae and Anthocerotae. The Journal of the Hattori Botanical Laboratory 68: 1–192.

[B16] Schliakov RN (1981) Pechenochnye Mkhi Severa SSSR. 4. Nauka, Leningrad, 221 pp.

[B17] Schuster RM (1974) The Hepaticae and Anthocerotae of North America East of the Hundredth Meridian. Vol. 3. Columbia University Press, New York and London, 880 pp.

[B18] Shaw B Crandall-Stotler B Váňa J Stotler RE von Konrat M Engel JJ Davis EC Long DG Sova P Shaw AJ (2015) Phylogenetic Relationships and Morphological Evolution in a Major Clade of Leafy Liverworts (Phylum Marchantiophyta, Order Jungermanniales): Suborder Jungermanniineae. Systematic Botany 40(1): 27–45. 10.1600/036364415X686314

[B19] Söderström L Hagborg A von Konrat M Bartholomew-Began S Bell D Briscoe L Brown E Cargill DC Costa DP Crandall-Stotler BJ Cooper ED Dauphin G Engel JJ Feldberg K Glenny D Gradstein SR He X Heinrichs J Hentschel J Ilkiu-Borges AL Katagiri T Konstantinova NA Larraín J Long DG Nebel M Pócs T Felisa PF Reiner-Drehwald E Renner MAM Sass-Gyarmati A Schäfer-Verwimp A Moragues JGS Stotler RE Sukkharak P Thiers BM Uribe J Váňa J Villarreal JC Wigginton M Zhang L Zhu RL (2016) World checklist of hornworts and liverworts. PhytoKeys 59: 1–828. 10.3897/phytokeys.59.6261 PMC4758082 26929706

[B20] Stephani F (1901) Species Hepaticarum 2. Bulletin de l’herbier Boissier, série 2. 1: 140–177.

[B21] Váňa J Söderström L Hagborg A von Konrat M Engel JJ (2010) Early land plants today: Taxonomy, systematics, and nomenclature of Gymnomitriaceae. Phytotaxa 11(1): 1–80. 10.11646/phytotaxa.11.1.1

[B22] Yamada K Choe DM (1997) A checklist of Hepaticae and Anthocerotae in the Korean peninsula. The Journal of the Hattori Botanical Laboratory 81: 175–242.

[B23] Yamada K Iwatsuki Z (2006) Catalog of the hepatics of Japan Journal of the Hattori Botanical Laboratory 99: 1–106.

[B24] Zhu R-L So ML Ye L-X (1998) A synopsis of the hepatic flora of Zhejiang, China. The Journal of the Hattori Botanical Laboratory 84: 159–174.

